# Defect‐Engineered Multi‐Intermetallic Heterostructures as Multisite Electrocatalysts for Efficient Water Splitting

**DOI:** 10.1002/advs.202502244

**Published:** 2025-04-17

**Authors:** Xiang‐Feng Wu, Zi‐Yan Li, Hui Wang, Jun‐Chuan Wang, Guo‐Qiang Xi, Xiao‐Jin Zhao, Chen‐Xu Zhang, Wu‐Gang Liao, Johnny C. Ho

**Affiliations:** ^1^ School of Materials Science and Engineering Hebei Key Laboratory of Advanced Materials for Transportation Engineering and Environment Shijiazhuang Tiedao University Shijiazhuang 050043 P. R. China; ^2^ State Key Laboratory of Radio Frequency Heterogeneous Integration (Shenzhen University) College of Electronics and Information Engineering Shenzhen 518060 P. R. China; ^3^ Department of Materials Science and Engineering City University of Hong Kong Hong Kong SAR 999077 P. R. China; ^4^ State Key Laboratory of Terahertz and Millimeter Waves City University of Hong Kong Hong Kong SAR 999077 P. R. China; ^5^ Institute for Materials Chemistry and Engineering Kyushu University Fukuoka 816‐8580 Japan

**Keywords:** defect, heterostructure, multi‐intermetallic, multisite electrocatalyst, nanoporous metal, water splitting

## Abstract

Efficient water splitting for renewable hydrogen production requires the development of highly active and stable electrocatalysts. This study investigates the design and synthesis of defect‐engineered multimetallic heterostructures as advanced electrocatalysts for overall water splitting. A synergistic approach combining atomic‐scale defect engineering and multiphase heterostructures is employed to enhance catalytic activity. A series of highly porous intermetallic alloys (CoCuMoNi) with abundant defect sites are synthesized using a high‐temperature alloying‐dealloying technique. Due to the synergistic effect of multiphase interfaces, built‐in electric fields, and defect engineering, the CoCuMoNi catalyst exhibits excellent bifunctional activity for water splitting (Hydrogen evolution reaction: 14 mV@10 mA cm^−2^; Oxygen evolution reaction (OER): 211 mV@10 mA cm^−2^; Overall water splitting: 1.559 V@100 mA cm^−2^), with significantly enhanced activity compared to pure metals and conventional materials. Additionally, these structures demonstrate excellent stability and durability. Advanced characterization techniques and density functional theory (DFT) reveal that the formation of defect sites and heterojunctions not only induces electronic modulation but also enhances intermetallic interactions and charge transfer from Ni and Mo to Cu and Co, facilitating intermediate formation and transformation, thereby boosting intrinsic activity. This work highlights the potential of defect‐engineered multimetallic heterostructures as scalable and efficient electrocatalytic platforms, paving the way for their practical applications in clean energy technologies.

## Graphical abstract

A self‐supporting nanoporous multicomponent CoCuMoNi catalyst is synthesized. Through the synergistic effect of multiphase interfaces, built‐in electric fields, and defect engineering, the binding energy of key reaction intermediates in hydrogen evolution reaction (HER)/oxygen evolution reaction (OER) is modulated, achieving high activity and stability in water electrolysis.

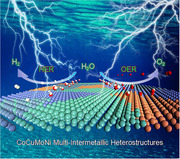

## 1 Introduction

Hydrogen, known for its high energy density and pollution‐free nature, is considered an ideal alternative to fossil fuels.^[^ ^1^, ^2^ ^]^ To meet the growing demand for large‐scale green hydrogen production and renewable energy storage, developing efficient, durable, and cost‐effective electrocatalysts is essential.^[^ ^3^ ^]^ Electrochemical water splitting, powered by renewable sources like solar and wind energy, offers an eco‐friendly and efficient approach to producing high‐purity green hydrogen.^[^ ^4^ ^]^ Although noble metal‐based catalysts, such as Ir and Pt, exhibit excellent activity in hydrogen evolution reaction (HER) and oxygen evolution reaction (OER), their high cost and low abundance hinder large‐scale applications.^[^ ^5^, ^6^ ^]^

To develop more cost‐effective electrocatalysts and accelerate the industrialization of hydrogen energy, researchers have devoted considerable effort to designing and developing non‐precious metal bifunctional catalysts that combine high catalytic performance and economic benefits. According to previous reports, these catalysts are typically based on nickel‐based materials, which are abundant on Earth and exhibit good water electrolysis performance similar to that of precious metals (such as Pt, Ru, etc.).^[^ ^7^, ^8^ ^]^ Examples include nickel‐based solid solution alloys,^[^ ^9^, ^10^ ^]^ compounds,^[^ ^11^, ^12^, ^13^ ^]^ or their mixtures.^[^ ^14^, ^15^, ^16^, ^17^ ^]^ Compared to single‐metal Ni, most of these materials exhibit varying degrees of enhanced alkaline HER/HOR electrocatalytic activity. However, these solid solution alloys often encounter element distribution issues and are thermodynamically prone to coarsening through low‐barrier atomic diffusion.^[^ ^18^ ^]^ Compared to single‐metal or bimetallic catalysts, medium‐entropy alloys, and high‐entropy alloys can provide different desired active sites and nearly continuous binding energy distributions through multi‐component design and multi‐element regulation, effectively stabilizing all intermediates during multi‐step catalytic reactions, thereby achieving good activity, selectivity, and stability.^[^ ^19^, ^20^ ^]^ Compared to disordered alloys (such as solid solutions), intermetallic heterostructures can not only generate electronic redistribution and achieve synergistic effects at the interfaces by combining different components but also create new interface structures by altering the composition and crystal phases of the structure, thereby realizing efficient overall water splitting catalytic functionality.^[^ ^21^, ^22^ ^]^ By mixing multiple elements and controlling their content, phase separation can be formed, leading to the generation of multi‐intermetallic heterostructures. These compounds typically exhibit long‐range disorder and short‐range order structures, forming heterogeneous interfaces composed of different elements at the nanoscale. The introduction of multiple elements creates numerous heterogeneous interfaces, typically the catalytic active centers, ultimately forming an interfacial activity “cocktail effect.”

Numerous studies have shown that local defects can modulate the electronic structure of electrocatalysts without compromising their structural integrity,^[^ ^23^, ^24^, ^25^ ^]^ thereby improving their electrocatalytic activity.^[^ ^26^ ^]^ The presence of defects induces electron delocalization, which increases the conductivity and charge density around the active sites.^[^ ^27^, ^28^ ^]^ At the same time, the coordination environment of the active sites can be adjusted by the presence of defects, increasing their exposure and active surface area, thus accelerating the interaction between reaction intermediates and catalytic sites.^[^ ^23^ ^]^

Combining the advantages of intermetallic heterostructures and local defects in electrocatalysis could significantly enhance water electrolysis performance. To verify this concept, we report a self‐supporting nanoporous hybrid electrode prepared using a physical metallurgy alloy‐dealloying strategy. This demonstrates excellent bifunctional performance in the water‐splitting electrocatalysis process. In this study, we selected a CoCuMoNi multicomponent intermetallic alloy. Due to the heterogeneous interfaces between the multicomponent metals and the built‐in electric fields generated by their electronegativity differences, the CoCuMoNi catalyst forms Co/Cu electrophilic regions and Mo/Ni nucleophilic regions, which respectively facilitate the adsorption of hydrogen and hydrogen‐oxygen intermediates in water splitting. Additionally, the lattice strain and distortion caused by grain boundaries also effectively modulate the binding energy of key reaction intermediates in HER/OER, significantly enhancing catalytic activity. In a 1.0 M KOH electrolyte, the nanoporous CoCuMoNi electrode requires only 14 mV (HER), 211 mV (OER), and 1.559 V (overall water splitting) overpotentials to reach current densities of 10, 10, and 100 mA cm^−2^, respectively, while maintaining excellent stability. This work provides a novel bifunctional multicomponent intermetallic heterostructure material for HER/OER, enabling the replacement of precious metals while maintaining high activity and stability in water electrolysis.

## 2 Results and Discussion

### 2.1 Materials Characterization

**Figure 1:**
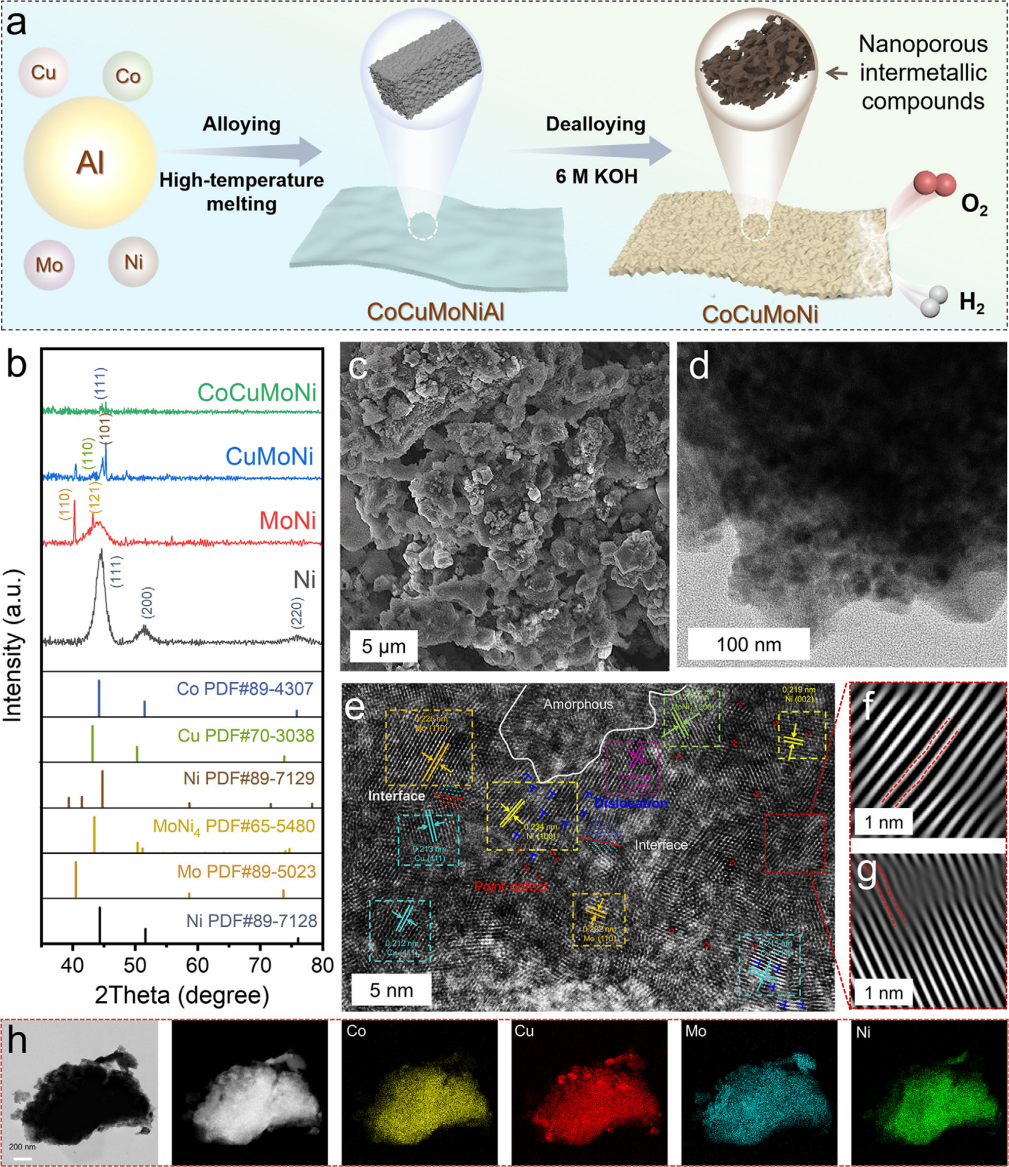
Microstructural properties. a) Synthesis diagram, b) XRD patterns, c) SEM, d) TEM, e) HRTEM image, f, g) Lattice fringes, h) HAADF‐STEM image and its corresponding EDS elemental mappings of CoCuMoNi.

To develop an alloy material with multiple grain boundaries, abundant defects, and a stable lattice structure, we considered the effects of intermetallic heterostructures and the electronegativity differences of elements. These factors help form a polycrystalline boundary structure and strong charge interactions, achieving excellent performance. Based on these considerations, we designed a self‐supporting 3D nanoporous CoCuMoNi hybrid electrode. A multi‐component structure was formed by controlling the feed ratio and constructing a polycrystalline boundary structure. The synthesis process is shown in **Figure** **1**a. First, a fully mixed precursor alloy of CoCuMoNiAl was formed by arc‐melting a mixture of five different transition metals (Co, Cu, Mo, Ni, Al) in an argon atmosphere. During the cooling process, an eutectic solidification reaction occurred, resulting in a bulk nanoporous structure composed of mainly insoluble mixed components. Then, selective etching was performed to remove the Al element through a chemical dealloying process in a 6 m KOH solution, ultimately forming the self‐supporting 3D nanoporous CoCuMoNi catalyst. We performed an Inductively Coupled Plasma Optical Emission Spectroscopy (ICP‐OES) analysis on the CoCuMoNi catalyst (Figure S1, Supporting Information), demonstrating that the residual aluminum content was as low as 5%. These results confirm that aluminum has been effectively removed to a significant extent during the de‐alumination process. The trace amount of residual aluminum could be attributed to delicate pores or aluminum being encapsulated by other metals, which hinders its complete removal. However, such minor residual aluminum does not negatively affect the catalytic activity of the system.

The detailed crystal structures of the synthesized Ni, MoNi, CuMoNi, and CoCuMoNi alloys were revealed through X‐ray diffraction (XRD). As shown in Figure 1b, diffraction peaks at 44.6°, 51.4°, and 75.8° correspond to the (111), (200), and (220) crystal planes of cubic Ni (PDF#89‐7128), respectively. After adding Mo, in addition to detecting the (110) crystal plane of cubic Mo (PDF#89‐5023) at 40.3°, the (121) crystal plane of tetragonal MoNi_4_ (PDF#65‐5480) was also observed at 43.2°. This indicates the formation of MoNi_4_ alloy during the mixing of Ni and Mo. With the addition of Cu, the (110) crystal plane of cubic Cu (PDF#70‐3038) at 43.3° was detected, while Ni underwent partial phase transformation, corresponding to the (002) plane of hexagonal Ni (PDF#89‐7129) at 45.3°. This phase transition is attributed to the structural distortion of Ni caused by the large Cu atoms, resulting in a displacement‐type transformation. With the addition of Co, the (111) crystal plane of cubic Co (PDF#89‐4307) at 44.2° was detected in the overall structure. We have added a magnified version of the XRD spectrum of the final CoCuMoNi alloy sample in Figure S2 (Supporting Information). The XRD spectrum of the CoCuMoNi alloy sample reveals that the crystallization peaks are weak and gradually shift toward amorphization. However, we can still distinguish the crystallization peaks corresponding to Ni, Mo, MoNi₄, Cu, and Co, indicating that the sample is partially crystalline. XRD testing confirmed that the prepared CoCuMoNi alloy comprises a multi‐intermetallic heterostructure material of Ni, Mo, MoNi_4_, Cu, and Co. Furthermore, a deeper analysis of the XRD data revealed that the intensity of the diffraction peaks gradually decreased with the increasing number of metal species. The broadening and right‐shifting of the diffraction peaks are mainly attributed to the ultrasmall crystal size and lattice contraction induced by the various transition metals. This suggests that introducing multiple elements may have increased defects within the alloy, driving it toward an amorphous phase.

Figure 1c,d displays representative scanning electron microscopy (SEM) and transmission electron microscopy (TEM) images of the nanoporous CoCuMoNi electrode, showing a hierarchical nanoporous structure composed of bulk particles with sizes of ≈2 µm and 20 nm. SEM (Figure S3, Supporting Information) and TEM (Figure S4, Supporting Information) images of Ni, MoNi, and CuMoNi reveal that these materials are composed of bulk particles with sizes of ≈4, 1, and 2 µm, respectively. This result is attributed to the selective etching of Al and the multiphase structure. Furthermore, in the TEM images (Figure S5, Supporting Information), a porous structure of ≈5 nm is clearly observed. Upon closer inspection, it was found that the particle size in the Ni sample was relatively larger due to the presence of only metallic Ni without the formation of other phase structures.Figure 1e presents a high‐resolution TEM (HRTEM) image of the nanoporous CoCuMoNi electrode. In the image, lattice fringes of the multiphase structure are observable. Analysis reveals that the crystal planes correspond to cubic Ni (100) (0.234 nm), Mo (111) (0.229 nm), MoNi_4_ (200) (0.201 nm), Cu (111) (0.210 nm), and Co (111) (0.206 nm). A detailed analysis of the HRTEM reveals numerous lattice distortions that lead to defects, including point defects, line defects, and amorphous regions. Additionally, significant crystal plane distortion is observed in the magnified regions (Figure 1f,g), which are caused by the disordered distribution of atoms at equivalent sites. This feature is also indicative of the gradual transition to a high‐entropy structure. A comparison of the HRTEM images and corresponding strain distribution maps (Figure S5, Supporting Information) of Ni, MoNi, CuMoNi, and CoCuMoNi shows that the lattice of the Ni sample is relatively orderly, with fewer and less intense strain regions. As the number of metal elements increases, the crystal plane area gradually decreases, defects increase, and strain intensifies. These lattice defects provide abundant low‐coordination sites to enhance catalytic activity.^[^ ^29^ ^]^ Moreover, heterogeneous interfaces between the phases were observed. These grain boundaries represent stable forms of crystal defects on the metal surfaces. The lattice strain and distortion induced by grain boundaries can also modulate the binding energy of key reaction intermediates.^[^ ^30^ ^]^ Scanning transmission electron microscopy (STEM) images and their corresponding energy‐dispersive X‐ray spectroscopy (EDS) elemental mapping images reveal a uniform distribution of each element (Figure 1h; Figure S6, Supporting Information). EDS (Figures S7 and S8, Supporting Information) confirms the atomic ratio of Co, Cu, Mo, and Ni in CoCuMoNi to be 23:19:44:14. At the same time, MoNi and CuMoNi have atomic ratios of 47:53 (Mo:Ni) and 75:5:20 (Cu:Mo:Ni), respectively. The significant differences in atomic ratios may be due to the local inhomogeneity of the multi‐intermetallic heterostructures. Therefore, TEM‐STEM‐EDS‐Mapping tests at different positions of CoCuMoNi were conducted (Figures S9 and S10, Supporting Information). The results confirm that the atomic composition varies at different locations, which leads to different catalytic activities at different positions of the multi‐intermetallic heterostructures for each reaction step, thereby displaying synergistic effects that enhance catalytic performance. We have replicated the synthesis of CoCuMoNi and performed additional TEM‐Mapping tests (Figure S11, Supporting Information) to demonstrate the reproducibility of the catalyst preparation. The HRTEM results (Figure S11a,b, Supporting Information) show that consistent with previous results, the CoCuMoNi catalyst retains a long‐range disordered and short‐range ordered state. It is composed of polycrystalline boundaries and multi‐intermetallic heterostructures. This confirms that the synthesis process is reproducible and that the catalyst composition is controllable across multiple preparations.

### 2.2 Electronic Property

**Figure 2:**
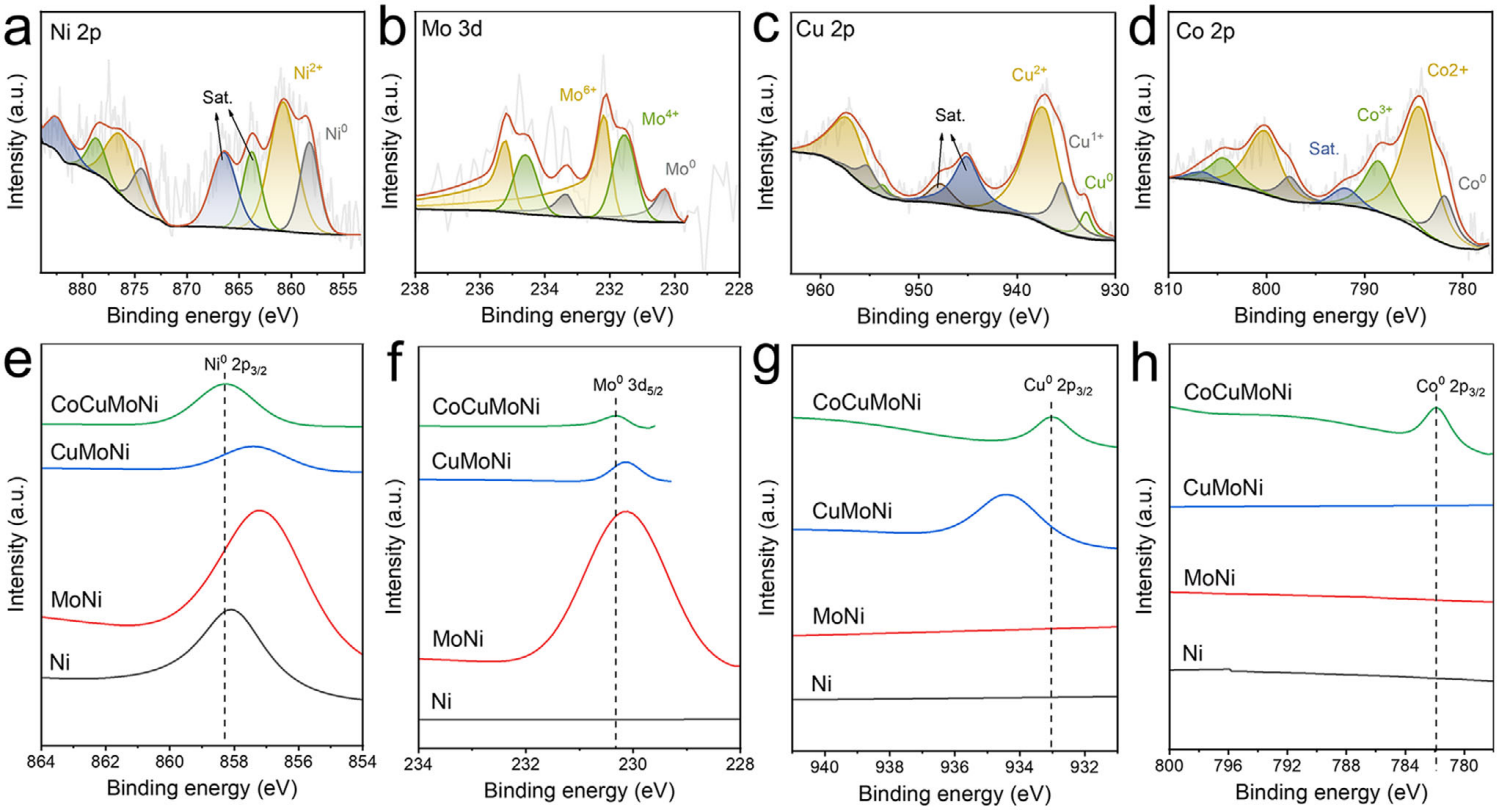
Chemical characterization. XPS spectra of a) Ni 2p, b) Mo 3d, c) Cu 2p, and d) Co 2p of CoCuMoNi. XPS spectras of e) Ni^0^ 2p_3/2_, f) Mo^0^ 3d_5/2_, g) Cu^0^ 2p_3/2_ and h) Co^0^ 2p_3/2_ of different samples.

The chemical states and electron transfer in the nanoporous CoCuMoNi electrode and control samples were further investigated using X‐ray photoelectron spectroscopy (XPS). As shown in Figure S12 (Supporting Information), the full XPS spectra of all samples indicate the coexistence of various elements in Ni, MoNi, CuMoNi, and CoCuMoNi. Further analysis of the high‐resolution XPS spectra for Ni 2p, Mo 3d, Cu 2p, and Co 2p in CoCuMoNi was conducted. In the Ni 2p XPS spectrum (**Figure** **2**a, Supporting Information), two characteristic peaks at binding energies of 858.3 and 860.5 eV correspond to Ni^0^ and Ni^2+^, respectively.^[^ ^31^, ^32^ ^]^ For the Mo 3d XPS spectrum (Figure 2b, Supporting Information), characteristic peaks at 230.3, 231.6, and 232.2 eV are attributed to Mo⁰, Mo^4+^, and Mo^6+^.^[^ ^31^ ^]^ As shown in Figure 2c, the Cu 2p spectrum, aside from the satellite peaks, can be deconvoluted into Cu^0^, Cu^1+^, and Cu^2+^ at binding energies of 933.0, 935.5, and 937.5 eV, respectively.^[^ ^33^ ^]^ In the Co 2p XPS spectrum (Figure 2d), the presence of Co^0^, Co^2+^, and Co^3+^ is observed, with binding energies of 781.9, 784.5, and 788.7 eV, respectively.^[^ ^32^, ^34^ ^]^ Figure 2a–d show the presence of Ni⁰, Mo⁰, Cu⁰, and Co⁰ in the CoCuMoNi electrode, confirming the metallic states of these elements in the material. In addition, small amounts of oxide species, which are inevitably generated due to surface oxidation or hydroxyl adsorption, are also observed.

Additionally, the XPS spectra of Ni 2p for the Ni electrode (Figure S13, Supporting Information), Ni 2p and Mo 3d for the MoNi electrode (Figure S14, Supporting Information), and Ni 2p, Mo 3d, and Cu 2p for the CuMoNi electrode (Figure S15, Supporting Information) were measured. The XPS spectra of Ni^0^, Mo^0^, Cu^0^, and Co° for the different electrodes were compared and analyzed (Figure 2e–h).Figure 2e compares the Ni^0^ XPS spectra of the different nanoporous electrodes. It is clear that the characteristic peak of MoNi (857.2 eV) shifts to a lower binding energy relative to Ni (858.1 eV). With the addition of Cu, the binding energy of Ni⁰ in the CuMoNi electrode gradually increases to 857.4 eV. After Mo is introduced, the electrons in the MoCuMoNi electrode transfer from the less electronegative Ni (858.3 eV) to the more electronegative Co. These phenomena suggest that the addition of Mo, Cu, and Co, along with the formation of Mo‐Ni, Cu‐Ni, and Co‐Ni covalent bonds, causes electron transfer from the less electronegative Mo to the more electronegative Ni, followed by transfer to the even more electronegative Cu and Mo. A similar trend is observed in the Mo^0^ (Figure 2f) and Cu^0^ XPS (Figure 2g) spectra. In the Mo^0^ XPS spectrum, the characteristic peak of the CuMoNi electrode (230.1 eV) does not shift relative to MoNi (230.1 eV). However, with the introduction of Co, the binding energy of Mo^0^ increases to 230.3 eV. In the Cu^0^ XPS spectrum, the characteristic peak of the MoCuMoNi electrode (933.0 eV) shifts noticeably to a lower binding energy than the CuMoNi electrode (934.5 eV). These phenomena indicate that with the addition of Co and the formation of Co‐Mo and Co‐Cu covalent bonds, electrons in the less electronegative Mo are transferred to the more electronegative Co and subsequently to the even more electronegative Cu. From the analysis above, it is clear that the differences in electronegativity between the elements and the construction of heterojunctions lead to significant charge transfer at the interfaces of the heterojunctions. Overall, Ni and Mo lose electrons, while Cu and Co gain electrons, resulting in a structure with high strength and activity. That is to say, the heterojunctions between the multi‐metallic components in the CoCuMoNi catalyst can generate charge interactions, leading to strong coupling effects and robust bonding, which promote the transfer of surface electrons.^[^ ^35^ ^]^ These interactions enhance the mechanical strength and structural stability of the catalyst. The multi‐component heterojunction interfaces provide distinct active sites and nearly continuous binding energy distributions, which can simultaneously stabilize all intermediates in multi‐step catalytic reactions.^[^ ^29^ ^]^ These high‐density heterojunction interfaces exhibit excellent synergistic effects, improving the overall electrolysis activity.

### 2.3 HER Performance

**Figure 3:**
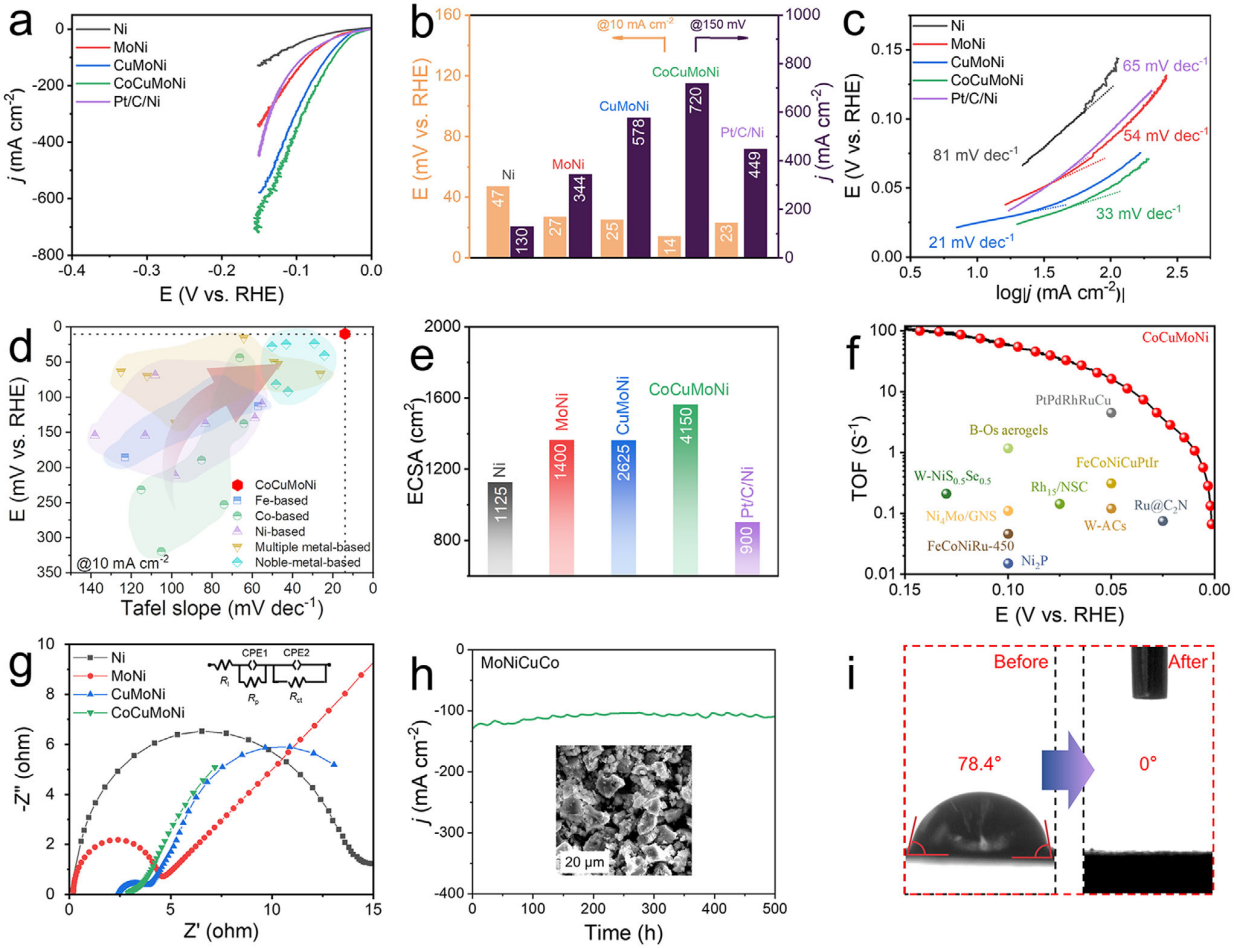
Electrochemical characterization of HER properties. a) Polarization curves, b) corresponding overpotentials, and c) Tafel slopes of various samples in 1 m KOH solution. d) Overpentials at 10 mA cm^−2^ for the as‐synthesized CoCuMoNi electrocatalyst, compared with reported electrocatalysts. e) ECSA histograms of different samples. f) TOF performance of the as‐synthesized CoCuMoNi electrocatalyst in comparison with reported related electrocatalysts. g) EIS curves of different samples. h) Constant current stability test of CoCuMoNi at 100 mA cm^−2^ for 500 h, with an SEM image inset showing the sample after the stability test. i) Contact angle images of CoCuMoNi before and after the stability test.

To evaluate the potential electrocatalytic ability of the fabricated nanoporous multi‐intermetallic heterostructures, the HER electrocatalytic activity of the CoCuMoNi electrode was first investigated using a standard three‐electrode configuration in nitrogen‐saturated 1 m KOH solution. **Figure** **3**a displays the typical HER polarization curve of the CoCuMoNi electrode after iR correction, compared with nanoporous MoNi, CuMoNi, bare Ni electrodes, and a commercial 20% Pt/C catalyst fixed on a nanoporous Ni current collector (Pt/C/Ni). It is evident that the CoCuMoNi electrode exhibits the best catalytic activity, requiring only 14 mV of overpotential to achieve 10 mA cm^−2^ (η_10_), outperforming Ni (47 mV), MoNi (27 mV), CuMoNi (25 mV), and Pt/C/Ni (23 mV). However, the η_10_ of the Ni electrode sharply increases to 47 mV, 33 mV higher than the CoCuMoNi electrode, indicating that Mo is a potential active site under alkaline conditions. Similar mechanisms have also been observed in earlier studies on the transition metal Mo, where it was found to have an electronic “regulation effect” on the active material (i.e., Ni) during the electroreduction process.^[^ ^36^ ^]^ In addition, part of the activity originates from the formation of the intermetallic compound MoNi₄, which has been shown to exhibit excellent catalytic activity in HER.^[^ ^31^ ^]^ Additionally, the MoNi₄ metal particles generated by the alloying of Ni and Mo were confirmed to play a significant role in promoting HER activity.^[^ ^31^, ^37^ ^]^ Impressively, the CoCuMoNi electrode exhibits industrial‐level current density (720 mA cm^−2^) at a small overpotential of 150 mV, which is ≈5.5 times, ≈2.1 times, ≈1.2 times, and ≈1.6 times higher than Ni (130 mA cm^−2^), MoNi (344 mA cm^−2^), CuMoNi (578 mA cm^−2^), and Pt/C/Ni (449 mA cm^−2^), respectively (Figure 3b). This significant enhancement in HER performance can be attributed to the formation of multi‐intermetallic heterostructures, which generate polycrystalline boundaries and defect sites that serve as HER catalytic active sites, thereby facilitating water adsorption/dissociation and H^*^ binding. As a result, the nanoporous CoCuMoNi electrode demonstrates markedly enhanced HER electrocatalytic performance. In contrast to MoNi, the HER activities of CuMoNi and CoCuMoNi electrodes are almost identical after the introduction of Cu and Co metals. This is because Cu and Co received substantial electrons from MoNi, which are then utilized for the HER reaction, which is consistent with the earlier XPS analysis. For HER, Cu and Co perform similar catalytic roles. The Tafel slope is an important indicator for evaluating the HER kinetics and catalytic activity.^[^ ^38^, ^39^, ^40^ ^]^ As shown in Figure 3c, the nanoporous CuMoNi and CoCuMoNi electrode exhibits a Tafel slope of only 21 and 33 mV dec^−1^, which is lower than that of Ni (65 mV dec^−1^), MoNi (54 mV dec^−1^), and the commercial Pt/C/Ni catalyst (65 mV dec^−1^).^[^ ^41^ ^]^ This result reflects the excellent Volmer‐Tafel reaction mechanism of the CoCuMoNi electrode. A lower Tafel slope indicates faster reaction kinetics and suggests that the CoCuMoNi electrode accelerates the rate‐determining step of the HER, likely through an efficient electron transfer process. To further evaluate the outstanding activity of the nanoporous CoCuMoNi electrode in HER, a comprehensive summary and comparison of the most recent state‐of‐the‐art catalysts in this field have been presented (Figure 3d; Table S1, Supporting Information), including Fe, Co, Ni, multi‐metallic, and noble metal‐based catalysts. The analysis shows that the nanoporous CoCuMoNi electrode exhibits outstanding HER activity and a low Tafel slope.

Electrochemical surface area (ECSA) is a critical parameter for characterizing the intrinsic density of active surface sites in catalysts. The ECSA values of Ni, MoNi, CuMoNi, and CoCuMoNi were determined via cyclic voltammetry (CV) measurements in the non‐Faradaic region at varying scan rates (Figure S16, Supporting Information). Compared to Ni (1 125 cm^2^), MoNi (1400 cm^2^), CuMoNi (2 625 cm^2^), and Pt/C/Ni (900 cm^2^), the CoCuMoNi electrode exhibited a significant increase in ECSA (4 150 cm^2^) (Figure 3e). This enhancement demonstrates that heterojunction interface engineering and defect engineering effectively increase the number of surface‐active sites. Based on the CoCuMoNi electrode's mass loading (2.72 mg cm^−2^), the corresponding mass activity was calculated to be ≈265 mA mg^−1^ (Figure S17, Supporting Information). To exclude the effects of structural and geometric changes and to highlight the intrinsic catalytic activity of the CoCuMoNi electrode, the HER linear sweep voltammetry (LSV) polarization curves were further normalized by the calculated ECSA (Figure S18, Supporting Information). The results indicate that the CoCuMoNi electrode requires only 47 mV to achieve a current density of 0.01 mA cm^−2^ ECSA, significantly lower than Ni (68 mV) and comparable to MoNi (49 mV) and CuMoNi (44 mV). It is worth noting that while Pt/C/Ni exhibits higher inherent activity, both CuMoNi and CoCuMoNi catalysts still show commendable inherent activity. This indicates that the CoCuMoNi catalyst, even without using precious metals, not only increases the active surface area but also demonstrates inherent activity comparable to Pt/C. This suggests that constructing multi‐component heterojunctions and forming multiple interfaces in the CoCuMoNi electrode enhances HER kinetics. The turnover frequency (TOF) values provide a means of comparing the activity of individual active sites in the catalyst. In alkaline HER, the TOF values of the nanoporous CoCuMoNi electrode at overpotentials of 25, 50, 75, 100, and 130 mV were 3.61, 16.39, 34.97, 57.79, and 91.43 s^−1^, respectively. These values exceed those of recently reported catalysts, further confirming the high intrinsic activity of the CoCuMoNi electrode (Figure 3f; Figure S19, Table S2, Supporting Information).

Electrochemical impedance spectroscopy (EIS) was employed further to investigate the reaction kinetics of the nanoporous CoCuMoNi electrode. As shown in Figure 3g, the nanoporous electrode exhibits two characteristic semicircles in the low‐to‐mid frequency range, corresponding to the interface charge transfer resistance (*R*_ct_) and pore resistance (*R*_p_) of the constant phase element (CPE). At higher frequencies, the real axis intercept represents the inherent resistance (*R*_I_) between the electrode material and the electrolyte. The equivalent circuit constructed using these specific descriptors is shown in the inset of Figure 3g. Compared to Ni (≈10.1, ≈63.94 Ω) and MoNi (≈4.1, ≈37.6 Ω), the *R*_ct_ and *R*_p_ values of CuMoNi (≈1.7, ≈11.18 Ω) and CoCuMoNi (≈1.4, ≈19.6 Ω) electrodes were significantly reduced (Figure S20, Supporting Information). The *R*_I_ values of CuMoNi and CoCuMoNi electrodes were ≈2.3 and 2.8 Ω, respectively, slightly higher than those of Ni (≈0.3 Ω) and MoNi (≈0.2 Ω). These results indicate that the multi‐intermetallic heterostructures have minimal impact on the system's inherent resistance, likely due to the stable structure induced by the formation of multi‐heterojunction interfaces. As a result, the system exhibits rapid interfacial charge transfer, consistent with the XPS results. In addition, contact angle measurements were performed to analyze the hydrophilicity of the hierarchical porous structures quantitatively. Compared to Ni (≈98.4°) and MoNi (≈87.3°), the contact angles of the CuMoNi (≈79.4°) and CoCuMoNi (≈78.4°) electrodes were significantly reduced (Figure S21, Supporting Information). This result indicates a marked improvement in the wettability of the CuMoNi and CoCuMoNi electrodes. It further demonstrates that the hierarchical porous structure formed after dealloying effectively enhances the contact area, providing many active sites for electrocatalytic reactions.^[^ ^42^ ^]^

The nanoporous CoCuMoNi electrode underwent stability testing for over 500 h at a current density of 100 mA cm^−2^ in a 1 M KOH electrolyte (Figure 3h). The results show that the nanoporous CoCuMoNi electrode retains exceptional stability. Post‐stability testing, the electrode was analyzed using SEM‐EDS‐Mapping. The results (Figure S22, Supporting Information) reveal that the overall structure of the nanoporous CoCuMoNi electrode remained largely unchanged, with a uniform distribution of Co, Cu, Mo, and Ni elements. We also performed an element leaching test by analyzing the electrolyte after 500 h of stability testing (Figure S23, Supporting Information). The results indicate that the leaching of various metal elements from the CoCuMoNi catalyst is minimal, with concentrations remaining at the µg/mL level. This confirms that the CoCuMoNi catalyst exhibits excellent resistance to leaching and further supports its outstanding stability. Furthermore, due to inevitable exposure to air after the stability test, some degree of oxidation occurred on the CoCuMoNi electrode. Notably, the nanoporous CoCuMoNi electrode exhibited outstanding hydrophilicity after the stability test. Full water wetting was achieved in just 0.16 s (Figure S24, Supporting Information). Compared to pre‐test conditions, the hydrophilicity of the CoCuMoNi electrode showed significant improvement (Figure 3i). This enhancement is likely due to surface activation during the electrocatalytic process and partial oxidation during the reaction.

### 2.4 OER Performance

**Figure 4:**
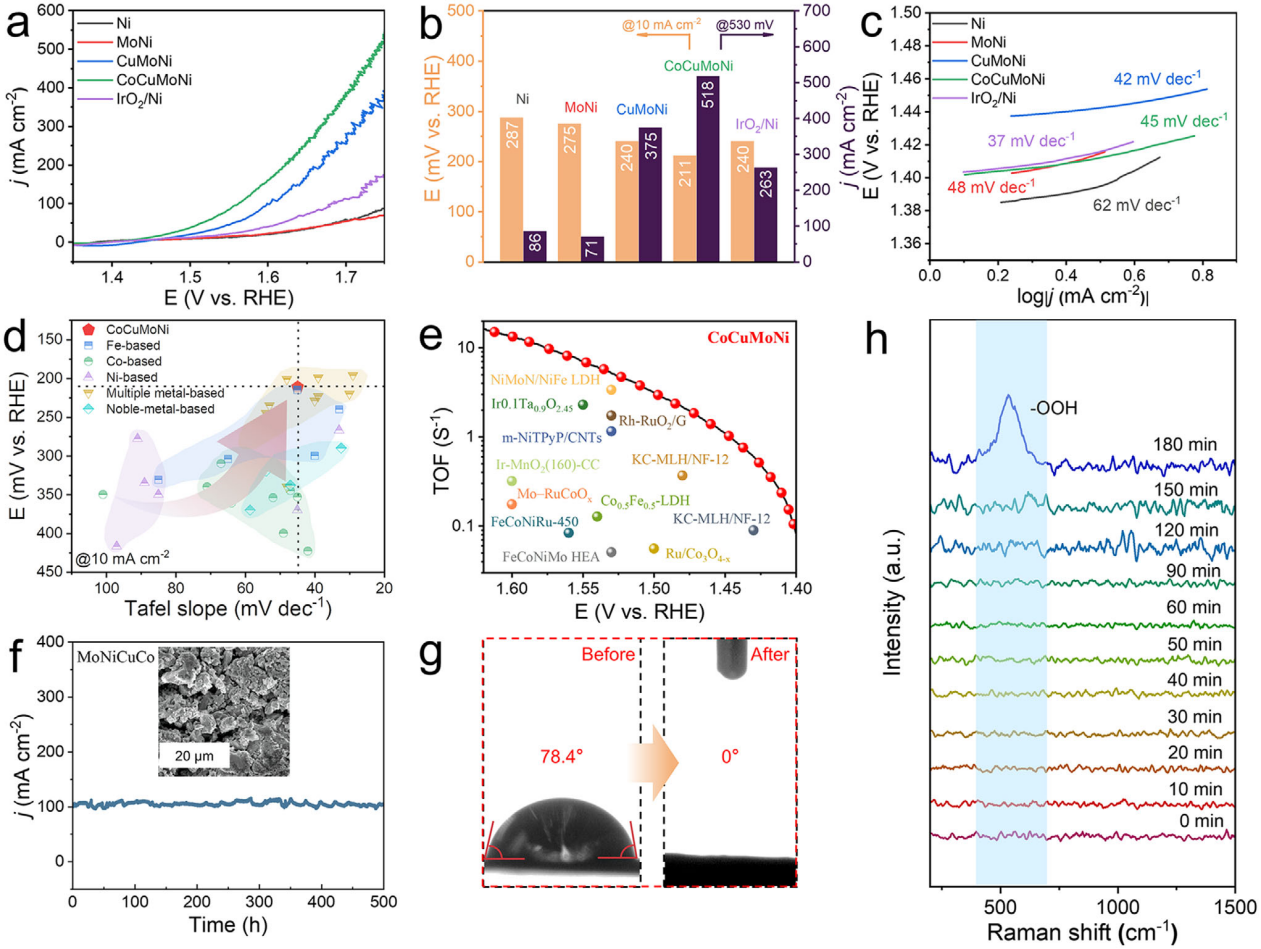
Electrochemical characterization of OER properties. a) Polarization curves, b) corresponding overpotentials, and c) Tafel slopes of various samples in 1 m KOH solution. d) Overpentials at 10 mA cm^−2^ and e) TOF performance of the as‐synthesized CoCuMoNi electrocatalyst compared with reported related electrocatalysts. f) Constant current stability test of CoCuMoNi at 100 mA cm^−2^ for 500 h, with an SEM image inset showing the sample after the stability test. g) Contact angle images of CoCuMoNi before and after the stability test. h) In situ Raman spectroscopy of CoCuMoNi during the stability test.

To investigate the role of the nanoporous CoCuMoNi electrode system in enhancing OER activity, the OER performance of MoNi, CuMoNi, CoCuMoNi, bare Ni electrodes, and commercial IrO₂ catalysts fixed on a nanoporous Ni current collector (IrO_2_/Ni) was studied under identical conditions (**Figure** **4**a). iR‐corrected LSV analysis showed that at a current density of 10 mA cm^−2^, the overpotential of the CoCuMoNi electrode was 211 mV (Figure 4b), significantly lower than those of Ni (287 mV), MoNi (275 mV), CuMoNi (240 mV), and commercial IrO_2_/Ni (240 mV). Notably, at 530 mV, the nanoporous CoCuMoNi electrode achieved an industrial‐grade current density of 518 mA cm^−2^, far exceeding that of commercial IrO_2_/Ni (263 mA cm^−2^). This indicates its superior efficiency at high current densities. To gain insight into the intrinsic kinetics of the nanoporous CoCuMoNi electrode system, the Tafel slope values were calculated. As shown in Figure 4c, the CuMoNi and CoCuMoNi electrodes exhibited the lowest Tafel slopes (42 and 45 mV dec^−1^) among all samples, significantly lower than those of Ni (62 mV dec^−1^) and MoNi (48 mV dec^−1^), and also outperforming commercial IrO_2_/Ni (37 mV dec^−1^). This confirms the synergistic effect promoted by the abundant heterojunction interfaces and defects in the CuMoNi and CoCuMoNi electrodes.^[^ ^43^ ^]^ This advantage is crucial, as it positions the electrode as a promising candidate for integration with intermittent and unstable power sources such as wind, tidal, and solar energy systems, thereby facilitating the production of green hydrogen. To further evaluate the OER performance achieved by the nanoporous CoCuMoNi catalyst, a summary and comparison of the most advanced electrocatalysts recently reported in the literature were made. As shown in Figure 4d and Table S3 (Supporting Information), the CoCuMoNi catalyst demonstrates excellent or comparable OER performance.

By calculating the TOF, the intrinsic activity of individual active sites in the CoCuMoNi electrode for the OER reaction was further determined. At overpotentials of 200, 250, 270, 300, 310, 320, 330, and 370 mV, the CoCuMoNi electrode exhibited maximum TOF values of 0.61, 2.18, 3.15, 5.23, 6.16, 7.06, 8.06, and 13.41 s^−1^, outperforming other recently reported catalysts (Figure 4f; Table S4 (Supporting Information). This indicates that the nanoporous CoCuMoNi electrode possesses outstanding intrinsic activity per unit site. In addition to electrocatalytic activity, a catalyst's long‐term stability and durability are crucial for industrial applications. The OER stability of the nanoporous CoCuMoNi electrode was assessed using chronoamperometry. As shown in Figure 4f, the CoCuMoNi electrode maintained stable performance for over 500 h at a current density of 100 mA cm^−2^, with negligible current decay, demonstrating remarkable structural stability. This is primarily attributed to the robust nanoporous structure and the excellent corrosion resistance of the multi‐intermetallic heterostructures.^[^ ^42^ ^]^ Moreover, after 500 h of continuous operation, SEM‐EDS‐Mapping of the CoCuMoNi electrode was performed, and as shown in Figure S25 (Supporting Information), the nanoporous structure of the electrode showed no significant changes, with the elements still uniformly distributed. We conducted an element leaching test by analyzing the electrolyte after subjecting the CoCuMoNi catalyst to 500 h of stability testing (Figure S26, Supporting Information). The results show that the leaching of metal elements from the catalyst is negligible, with concentrations remaining at the µg/mL level. This observation highlights the CoCuMoNi catalyst's exceptional resistance to leaching, further confirming its remarkable stability. This behavior is attributed to the intersection of polycrystalline boundaries, which form a network that effectively suppresses dislocation movement, stabilizing them within the bulk metal.^[^ ^44^ ^]^ As a result, the catalyst shows a high operational lifetime. Notably, the contact angle of the CoCuMoNi electrode after stability testing was 0° (Figure 4g), and the wetting process was achieved in ≈0.96 s (Figure S27, Supporting Information). This indicates that the CoCuMoNi electrode possesses a highly hydrophilic nanoporous surface during the OER process, which facilitates the infiltration of the electrolyte solution and promotes rapid reactions.

In the EDS results, we also observed that oxygen elements predominated in the composition. In addition to the partial oxidation of the CoCuMoNi electrode due to exposure to air, the main contribution to the oxygen content comes from the oxidation of the electrode's loose, porous surface during the OER process. The anodic oxidation of Co, Cu, Mo, and Ni metals typically generates the actual active species for OER.^[^ ^45^, ^46^ ^]^ To further investigate the surface modifications, we performed in situ Raman (Figure 4h; Figure S28, Supporting Information) and XPS (Figure S29, Supporting Information) measurements on the CoCuMoNi electrode after stability testing. The in situ Raman results showed that after 180 min of constant current stability testing, a characteristic M‐O vibration (M = Ni, Mo, Cu, Co) appeared ≈535 cm^−1^ corresponding to M‐OOH.^[^ ^47^ ^]^ This indicates that surface structural reconstruction occurred on the CoCuMoNi electrode during the OER process, forming a hydroxide oxide layer that facilitates the enhancement of OER activity.^[^ ^45^, ^46^ ^]^ Additionally, the O 1s spectra of the CoCuMoNi electrode surface were measured using a multi‐step etching module in XPS to reveal its near‐surface structure. The results showed that before etching, the O 1s peak at 537.6, 533.7, and 531.3 eV were deconvoluted into peaks corresponding to adsorbed H_2_O, surface hydroxyl (O‐H), and metal‐lattice oxygen (M‐O).^[^ ^48^, ^49^ ^]^ After multi‐step etching, the adsorbed water gradually disappeared, while O‐H and M‐O remained, with the M‐O peak occupying the largest area. This further confirms the oxidation and reconstruction of the surface structure of the CoCuMoNi electrode. The presence of O‐H and M‐O species indicates the dual activation of the CoCuMoNi electrode through AEM and LOM during the OER process.^[^ ^47^ ^]^ As shown in Figure S30a (Supporting Information), we conducted in situ overpotential‐resolved electrochemical infrared spectroscopy to identify reaction intermediates and to investigate the influence of steric interactions on the reaction mechanism during oxygen evolution. We observed distinct absorption peaks at 1022 and 1096 cm^−^¹ as the bias potential reached the polarization region. These peaks indicate the production of oxygen intermediates, such as *OOH, before the release of O₂. The observed hump is attributed to the hydrogen bonding of *OOH with neighboring species, which affects the molecular vibrations, as described in previous studies.^[^ ^50^, ^51^, ^52^ ^]^ Interestingly, we also found that the intensity of the vibrational band associated with the hydrogen bond increased as the reaction potential increased, which we interpret as indicating a stronger interaction with H on fully oxidized surfaces at high potentials, optimizing proton adsorption energy at the bridging oxygen sites. A peak at a vibration frequency of ≈1261 cm^−^¹ appeared at an applied potential of 1.33 V, which can be attributed to the stretching vibration of the *OO intermediate. A similar phenomenon was also observed in the in‐situ time‐resolved electrochemical infrared spectroscopy test (Figure S30b, Supporting Information).

### 2.5 Mechanistic Insights into Catalytic Activity

**Figure 5:**
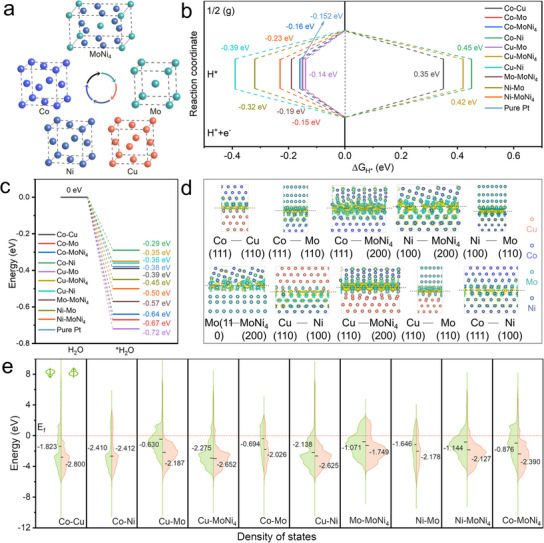
DFT characterization. a) Schematic illustration of multi‐component mixing in CoCuMoNi sample. b) The free energy distribution for HER, c) E_ads_(H_2_O), d) differential charge density diagrams, the darker yellow and cyan colors correspond to a stronger tendency for electron depletion and accumulation, respectively, and e) PDOS for different samples.

Density functional theory (DFT) calculations were performed to elucidate the structural origins of the superior water electrolysis activity of the nanoporous CoCuMoNi electrode. XRD and TEM results revealed that the CoCuMoNi system undergoes significant phase separation, forming a multi‐intermetallic heterostructure composed of Ni, Mo, MoNi_4_, Cu, and Co. This phase separation leads to the formation of numerous heterojunction interfaces between the different phases. Based on this, we used Ni (100), Mo (110), MoNi_4_ (200), Cu (110), and Co (111) as the building blocks and combined them pairwise to construct ten different heterojunction structures (**Figure** **5**a; Figure S31, Supporting Information), enabling a comprehensive investigation of the effects of each interface on the adsorption energies and electronic structures of the nanoporous CoCuMoNi electrode system. Additionally, the atomic structures of Ni (100), Mo (110), MoNi_4_ (200), Cu (110), and Co (111) were constructed and analyzed for comparison.

The rapid OER reaction is primarily attributed to hydroxide oxide active species, significantly enhancing the OER process. Therefore, we calculated the hydrogen adsorption Gibbs free energy (ΔG_H*_) for all heterostructures using DFT, as shown in Figure 5b. The trend in ΔG_H*_ values for the ten heterostructures is as follows: Cu‐Mo (−0.14 eV) > Co‐Mo (−0.15 eV) > Co‐MoNi_4_ (−0.16 eV) > Mo‐MoNi_4_ (−0.19 eV) > Ni‐MoNi_4_ (−0.23 eV) > Ni‐Mo (−0.32 eV) > Co‐Cu (0.35 eV) > Cu‐Ni (−0.39 eV) > Cu‐MoNi_4_ (0.42 eV) > Co‐Ni (0.45 eV). ΔG_H*_ of Pt reaches −0.152 eV. Comparing this to the CoCuMoNi catalyst, we found that the Cu‐Mo and Co‐Mo heterojunctions exhibit hydrogen adsorption Gibbs free energies that outperform Pt. In contrast, the Co‐MoNi₄ heterojunction is comparable to Pt in this regard. This indicates that the formation of Cu‐Mo, Co‐Mo, and Co‐MoNi₄ heterojunctions is more favorable for forming and releasing molecular hydrogen. A comparison of the ΔG_H*_ values between the heterojunction structures and pure metals clearly shows that the ΔG_H*_ of any heterojunction structure is superior to those of Ni (−0.51 eV), Mo (−0.55 eV), MoNi_4_ (−0.48 eV), Cu (0.61 eV), and Co (0.72 eV) (Figure S32, Supporting Information), demonstrating that the multi‐heterojunction interfaces formed in the CoCuMoNi electrode effectively promote the HER reaction. We also compared the H_2_O adsorption energies (E_ads_(H_2_O)) for various structures (Figure 5c; Figure S33, Supporting Information). The results revealed a trend similar to that of E_ads_(H_2_O) values: Cu‐Mo (−0.72 eV) < Co‐Mo (−0.67 eV) < Co‐MoNi_4_ (−0.64 eV) < Mo‐MoNi_4_ (−0.57 eV) < Ni‐MoNi_4_ (−0.50 eV) < Ni‐Mo (−0.45 eV) < Co‐Cu (−0.39 eV) < Cu‐Ni (−0.36 eV) < Cu‐MoNi_4_ (−0.35 eV) < Co‐Ni (−0.29 eV). Additionally, the E_ads_(H_2_O) on Pt reaches −0.38 eV. Co‐Ni, Cu‐MoNi₄, and Cu‐Ni heterojunctions demonstrate superior water adsorption energies, while the Mo‐MoNi₄ heterojunction shows a water adsorption energy similar to that of Pt. These results highlight the excellent catalytic properties of the CoCuMoNi catalyst in comparison to Pt, particularly under alkaline conditions. Notably, when compared to the E_ads_(H_2_O) values of Ni (−0.53 eV), Mo (−0.67 eV), MoNi_4_ (−0.94 eV), Cu (−0.15 eV), and Co (−0.48 eV), it is evident that the Ni, Mo, and MoNi_4_ components in the CoCuMoNi electrode favor H_2_O adsorption. Moreover, We have calculated the theoretical formation energies for the resulting structures. As shown in Figure S34 (Supporting Information), the formation energies of the heterojunctions are all negative, indicating that the obtained heterostructures are thermodynamically stable.

To further elucidate the origin of the high catalytic activity in the CoCuMoNi electrode, differential charge density and partial density of states (PDOS) calculations were performed. The differential charge density results (Figure 5d; Figure S35, Supporting Information) show significant charge interaction at the interfaces of the 10 heterojunctions, leading to enhanced structural stability and activity. This high‐density active site optimizes their electronic configurations and intermediate adsorption energies, improving their catalytic performance. A closer inspection of the data reveals that the Co‐Mo, Co‐MoNi_4_, Cu‐MoNi_4_, and Cu‐Mo heterojunctions exhibit the most substantial charge transfer.^[^ ^53^, ^54^, ^55^ ^]^ A deeper analysis, in conjunction with the XPS results, shows that this is due to the electronegativity differences between Cu/Co and Mo/MoNi_4_, which lead to the formation of dipoles at the interfaces. These dipoles establish charge transfer channels and exhibit a synergistic effect, optimizing the adsorption of active intermediates on the CoCuMoNi surface and promoting efficient HER reactions.^[^ ^56^ ^]^ Figure 5e displays the d‐band partial density of states (PDOS) for the 10 heterostructures in the CoCuMoNi electrode. The strong d‐d Coulomb interactions split the d‐orbitals into a filled bonding state with spin‐up and an empty anti‐bonding state with spin‐down. The anti‐bonding states above the Fermi level (E_f_) can describe the adsorption‐metal interaction, as the electrons filling the anti‐bonding states primarily influence the bond strength between the adsorbate's valence state and the transition metal's d‐states.^[^ ^26^ ^]^ We observed the following order for the anti‐bonding state centers: Cu‐Mo (−0.630 eV) > Co‐Mo (−0.694 eV) > Co‐MoNi_4_ (−0.876 eV) > Mo‐MoNi_4_ (−1.071 eV) > Ni‐MoNi_4_ (−1.144 eV) > Ni‐Mo (−1.646 eV) > Co‐Cu (−1.823 eV) > Cu‐Ni (−2.138 eV) > Cu‐MoNi_4_ (−2.275 eV) > Co‐Ni (−2.410 eV). This trend is consistent with that observed for ΔG_H*_. The anti‐bonding state centers of Cu‐Mo and Co‐Mo are closer to the E_f_, suggesting stronger interactions between the adsorbates and the active sites.^[^ ^26^ ^]^ Additionally, the continuous charge presence near the Ef for each heterojunction indicates that these structures possess excellent conductivity, facilitating charge transfer. Thus, the DFT results indicate that these multi‐heterojunction interfaces in the multi‐metallic compound create multiple sites to overcome linear catalytic activity and regulate the adsorption energies of water molecules and hydrogen intermediates. This provides an important multi‐interface engineering strategy for designing efficient water electrolysis catalysts.

### 2.6 Overall Water Splitting Efficiency

**Figure 6:**
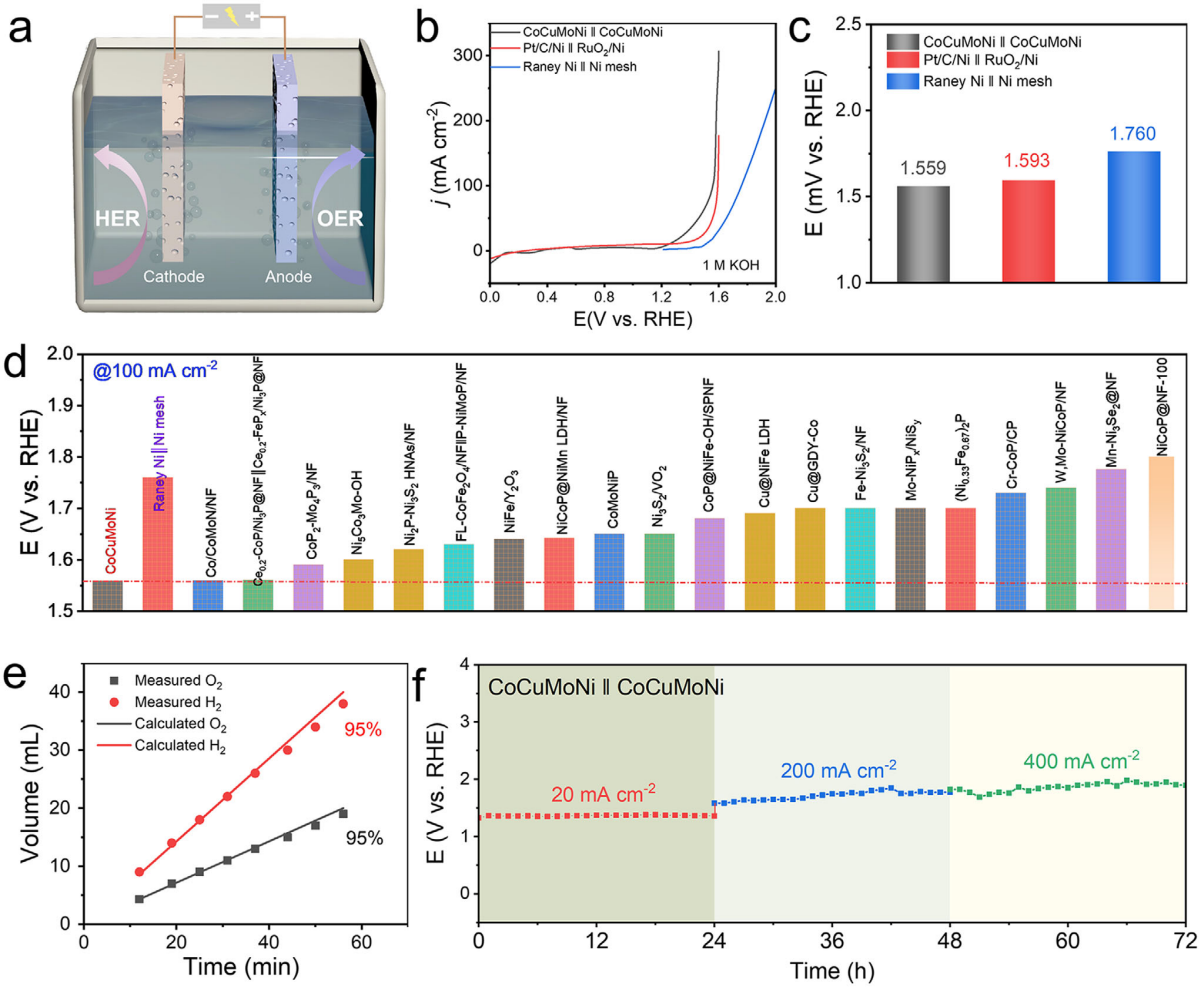
Electrochemical performance in alkaline water electrolyzer. a) Schematic diagram of the electrolyzer reaction device. b) Polarization curves, and c) corresponding overpotentials at 10 mA cm^−2^ for different samples in 1 m KOH aqueous electrolyte. d) Overpotentials of the as‐synthesized CoCuMoNi electrocatalyst at 100 mA cm^−2^ compared to reported related electrocatalysts. e) FE for H_2_ and O_2_ generation over the CoCuMoNi electrode at 10 mA cm^−2^ for 600 min. f) Constant current performance of CoCuMoNi || CoCuMoNi at 20, 200, and 400 mA cm^−2^ for 24 h, respectively.

Stability and durability are key concerns in industrial applications. Industrial water electrolysis cells operate under harsh conditions, requiring electrodes that can withstand high‐alkaline environments, high current loads, and intense mechanical stresses induced by bubble formation, thus preventing severe chemical, electrochemical, and mechanical corrosion.^[^ ^43^ ^]^ Given the excellent performance of the nanoporous CoCuMoNi electrode in both HER and OER, an overall water‐splitting electrolyzer was assembled, using it as both the cathode and anode to demonstrate its suitability for practical water‐splitting systems (**Figure** **6**a).^[^ ^26^ ^]^ The polarization curves of the CoCuMoNi‐based electrolyzer in 1 M KOH electrolyte are shown in Figure 6b, compared with commercial Pt/C/Ni || RuO_2_/Ni and Raney Ni || Ni mesh.^[^ ^57^ ^]^ Notably, at 10 mA cm^−2^, the CoCuMoNi electrode shows a low cell voltage of 1.559 V, outperforming Pt/C/Ni || RuO_2_/Ni (1.593 V) and Raney Ni || Ni mesh (1.760 V) (Figure 6c), as well as most recently reported bifunctional catalysts (Figure 6d; Table S5, Supporting Information). Moreover, the CoCuMoNi electrode exhibits excellent Faradaic efficiency (FE) for water electrolysis (Figure 6e; Figure S36, Supporting Information). In a 60‐minute test, the actual generation rates of H_2_ and O_2_ remained at ≈95%, and the H_2_/O_2_ generation ratio consistently stayed around 2:1, demonstrating excellent and stable durability. Therefore, the stability and durability of the CoCuMoNi electrode were further investigated using multi‐current‐step chronopotentiometry (Figure 6f). Current densities of 20, 200, and 400 mA cm^−2^ were applied, and the potential immediately responded and stabilized, remaining virtually unchanged over 24 h. This rapid response and quick potential stabilization with increasing current density indicate fast mass diffusion on the electrode surface. These characteristics enable the electrode to rapidly start up and respond in industrial water electrolysis cells, especially when powered by intermittent and unstable renewable energy sources such as wind, solar, and hydropower.^[^ ^26^, ^43^ ^]^ Given the economic advantages of using nonprecious metal elements, the simplicity of the fabrication method, scalability for mass production, and exceptional performance at industrial‐relevant current densities, the newly developed nanoporous CoCuMoNi multi‐intermetallic heterostructures hold great promise as a potential electrocatalyst for practical water splitting applications.

## 3 Conclusion

This study contributes significantly to the electrocatalysis field by demonstrating the design and synthesis of defect‐engineered multimetallic heterostructures for efficient and stable overall water splitting. Combining atomic‐scale defect engineering and multiphase heterostructures, we achieved a porous multi‐intermetallic heterostructure (CoCuMoNi) with abundant heterojunction interfaces and defect sites that deliver superior bifunctional activity. The materials exhibited low overpotentials for the HER (14 mV@ 10 mA cm^−2^) and OER (211 mV@ 10 mA cm^−2^) and efficient overall water splitting at 1.559 V for 100 mA cm^−2^. These metrics significantly outperform conventional electrocatalysts. Detailed analyses using advanced characterization techniques and DFT reveal that the defect sites and heterojunctions enhance electronic modulation, intermetallic interactions, and charge transfer dynamics, ultimately boosting catalytic performance. These findings validate the effectiveness of the proposed synergistic design strategy and highlight the transformative role of defect engineering in optimizing catalytic properties. The results of this study offer a potential solution for expanding the application of high‐performance electrocatalysts in clean energy.

## 4 Experimental Section

### Materials

Co (99.9%), Cu (99.9%), Mo (99.9%), Ni (99.9%), and Al (99.9%) were purchased from Beijing Zhongke Yannuo New Materials Technology Co., Ltd. Sodium hydroxide (NaOH, A.R.) was purchased from Honeywell International Inc. Pt/C (A.R.) and ruthenium dioxide (RuO_2_, A.R.) were purchased from Shanghai Aladdin Biochemical Technology Co., Ltd. Deionized (DI) water was obtained from the OmniaLab ED+40 ultrapure water system.

### Preparation of CoCuMoNi System Electrocatalysts

Alloy precursors with dimensions of 1 cm × 0.5 cm × 250 µm, including Ni_20_Al_80_, Mo_4_Ni_16_Al_80_, Mo_4_Cu_8_Ni_8_Al_80_, Mo_4_Co_5.3_Cu_5.3_Ni_5.3_Al_80_ (at%), were prepared by arc‐melting Ni and Al under an N_2_ atmosphere, with/without the addition of Co, Cu, or/and Mo, followed by furnace cooling and cutting procedures. These precursors were then immersed in a 6 m NaOH solution saturated with N_2_ and reacted for 4 h to chemically dealloy and remove Al. The dealloyed samples were rinsed with ultrapure water to remove residual chemicals from the nanopores, resulting in the preparation of nanoporous Ni, MoNi, CuMoNi, and CoCuMoNi. Pt/C/Ni and RuO_2_/Ni electrodes were prepared by mixing commercial Pt/C and RuO_2_ nanocatalysts with Nafion in a solution containing 20% ethanol and 80% water, followed by drop‐casting onto the nanoporous Ni skeletons prepared as described above.

### Characterization

Analyses such as TEM, HRTEM, and EDS were performed using a JEOL JEM‐2100F instrument. Surface morphologies were examined via SEM (S‐4800) with an EDS detector at acceleration voltages of 5 and 30 kV. The crystal structures of the alloy sheets were characterized using a D2 PHASER XE‐T X‐ray Diffractometer System, employing Cu Kα radiation (λ = 1.5406 Å). Raman spectra were acquired using a WITec alpha 300 R Raman System, excited by a 532 nm laser source. XPS measurements were carried out with a PHI 5000 Versaprobe system equipped with monochromatic Al Kα radiation, and the binding energies were calibrated by aligning the C 1s peak to 284.8 eV. Contact angles of electrode tabs were determined using a DataPhysics Contact Angle Tester. The contact angle measurements performed after the stability tests for HER and OER were conducted on dried catalyst substrates. The ICP‐OES test was conducted using the ICP‐OES6300 product from Thermo Fisher Scientific, USA. Infrared spectroscopy was tested using the PerkinElmer Fourier Transform Infrared Spectroscopy Spectrometer.

### Electrochemical Measurements

All electrochemical measurements were performed using a standard three‐electrode setup connected to a Chenhua CHI760E electrochemical workstation. To eliminate the passivation layer on the working electrode, 20 CV cycles were conducted at a scan rate of 5 mV s⁻¹ before LSV testing. In a 1 m KOH solution, a Hg/HgO electrode was the reference electrode, while a graphite rod was used as the counter electrode. In the overall water‐splitting experiment, the CoCuMoNi catalyst was used as both the anode and cathode. The anode was connected to the working electrode, while the cathode was connected to the counter and reference electrodes. LSV scans were performed at a scan rate of 5 mV s^−1^ for HER, OER, and overall water splitting, covering potential ranges of −1.6 to −0.9 V, 0 to 1.5 V, and 0 to 1.5 V, respectively. All potentials in 1 M KOH were converted to values versus the reversible hydrogen electrode (RHE) using Equation (1):

(1) $$E_{\mathrm{RHE}}=E_{\mathrm{Hg}/\mathrm{HgO}}+0.098+0.059\times \mathrm{pH}$$

where *E*_RHE_ and *E*_Hg/HgO_ are the potentials versus RHE and measured potentials versus Hg/HgO reference electrode, respectively.

The ECSAs of the catalysts were derived from the double‐layer capacitance (C_dl_) profiles using Equation (2):^[^ ^58^, ^59^ ^]^

(2) $$\mathrm{ECSA}=\left(C_{dl\;}/C_{s}\right)A_{geo}$$

where the specific capacitance (0.040 mF cm^−2^) and Ageo (0.5 cm^2^) represent the geometric surface area of the alloy sheets exposed to the electrolyte. Non‐Faradaic currents were first measured, and C_dl_ values were determined from the slope of the linear fit between the capacitive current and scan rate.^[^ ^60^ ^]^

CV scans were performed in 1 m phosphate‐buffered saline (PBS) to determine TOF at a scan rate of 50 mV s^−1^ over a potential range of −0.2 to 0.6 V (vs RHE). The TOF was calculated using Equations (3) and (4):^[^ ^61^ ^]^

(3) TOF=I/2nF

(4) n=Q/2F

where *I* is the current (in A), n is the number of active sites (in mol), F is the Faraday constant (96 500 C mol^−1^), and Q is the charge associated with voltammetric processes (in C).

EIS measurements were conducted at open‐circuit potential over a frequency range of 100 kHz to 0.01 Hz.

### Computational Methods

Electronic structure optimizations were performed using spin‐polarized DFT calculations, implemented through the plane‐wave method in the Vienna Ab Initio Simulation Package (VASP).^[^ ^62^ ^]^ The interactions between core and valence electrons were described using the projector augmented wave (PAW) method. At the same time, the generalized gradient approximation (GGA) with the Perdew‐Burke‐Ernzerhof (PBE) functional was employed to account for exchange‐correlation energies.^[^ ^63^ ^]^ A plane‐wave cutoff energy of 450 eV was applied, and spin‐polarization was considered for all structural relaxations. The optimization process adhered to a force convergence criterion of 0.05 eV Å^−1^ per atom, with a self‐consistent‐field (SCF) energy convergence threshold set at 10^−4^ eV.

The surface models of Co(111), Cu(111), Mo(110), MoNi_4_(200), and Ni(100) were constructed with a thickness of four atomic layers, and a vacuum layer of 15 Å was applied. The interface models were created by combining different supercells of metals. Specifically, the interface model of Ni‐MoNi_4_ was constructed by combining Ni (7 × 3 × 1 supercells) with MoNi_4_ (3 × 2 × 1). The Ni‐Mo interface model was built by combining Ni (7 × 4 × 1 supercells) with Mo (4 × 3 × 1 supercells). For the Mo‐MoNi_4_ interface, Mo (4 × 1 × 1 supercells) was combined with MoNi_4_ (3 × 1 × 1 supercells). The Cu‐Ni interface model was built by combining Cu (1 × 2 × 1 supercells) with Ni (2 × 7 × 1 supercells). Similarly, the Cu‐Mo interface was created by combining Cu (1 × 1 × 1 supercell) with Mo (1 × 3 × 1 supercell). The Cu‐MoNi_4_ interface was constructed by combining Cu (1 × 2 × 1 supercell) with MoNi_4_ (1 × 5 × 1 supercell). The Co‐Ni interface was built by combining Co (1 × 2 × 1 supercells) with Ni (2 × 7 × 1 supercells), and the Co‐MoNi_4_ interface model was created by combining Co (1 × 2 × 1 supercells) with MoNi₄ (1 × 5 × 1 supercell). The Co‐Mo interface was constructed using Co (1 × 1 × 1 supercell) and Mo (1 × 3 × 1 supercell), while the Co‐Cu interface model was built by combining Co (1 × 1 × 1 supercell) with Cu (1 × 1 × 1 supercell).

OER and HER free energy profiles for the FeCoNiWCuOOH system were derived, with the overall OER/HER reactions expressed through Equations (5)–(9):

For OER:

(5) $$4OH^{-}\to \;OH^{*}+3OH^{-}+e^{-}$$

(6) $$\to O^{*}+H_{2}O+2OH^{-}+2e^{-}$$

(7) $$\to \mathrm{HO}O^{*}+H_{2}O+OH^{-}+3e^{-}$$

(8) $$\to O_{2}+2H_{2}O+4e^{-}$$

For HER:

(9) $$H^{+}+e^{-}\to 1/2H_{2}$$

In equilibrium, the free energy of each hydrogen atom was assumed to be equal in the initial and final states. Free energies of the OER/HER intermediates were calculated using Equation (10):

(10) ΔG=ΔE+ΔZPE−TΔS

where ΔG, ΔS, and ΔZPE represent the binding energy, entropy change, and zero‐point energy change at 298.15 K, respectively, where ΔG is the Gibbs free energy, ΔE is the binding energy, ΔZPE is the zero‐point energy correction, and ΔS is the entropy change, evaluated at 298.15 K.^[^ ^64^ ^]^

## Conflict of Interest

The authors declare no conflict of interest.

## Supporting information



Supporting Information

## Data Availability

The data that support the findings of this study are available from the corresponding author upon reasonable request.
