# Supplementary material for: Defect‐Engineered Multi‐Intermetallic Heterostructures as Multisite Electrocatalysts for Efficient Water Splitting
Source: Adv Sci (Weinh). 2025 Apr 17;12(26):2502244. doi: 10.1002/advs.202502244 (PMC12245022; doi:10.1002/advs.202502244)
Supplement: Supplementary file 1 — Supporting Information [file ADVS-12-2502244-s001.pdf]

ADVANCED SCIENCE
Open Access

Supporting Information

for *Adv. Sci.*, DOI 10.1002/advs.202502244

Defect-Engineered Multi-Intermetallic Heterostructures as Multisite Electrocatalysts for Efficient Water Splitting

Xiang-Feng Wu, Zi-Yan Li, Hui Wang, Jun-Chuan Wang, Guo-Qiang Xi, Xiao-Jin Zhao, Chen-Xu Zhang, Wu-Gang Liao* and Johnny C. Ho**

Supporting Information

Defect-Engineered Multi-Intermetallic Heterostructures as Multisite Electrocatalysts for Efficient Water Splitting

Xiang-Feng Wu,[#] Zi-Yan Li,[#] Hui Wang, Jun-Chuan Wang, Guo-Qiang Xi, Xiao-Jin Zhao, Chen-Xu Zhang,[*] Wu-Gang Liao,[*] and Johnny C. Ho[*]

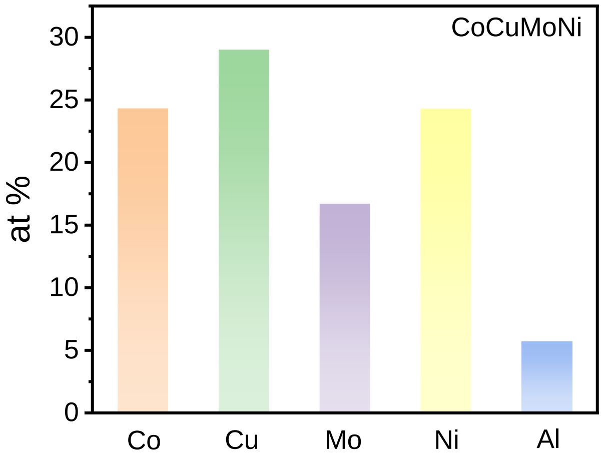

Figure S1. ICP spectra of CoCuMoNi after the chemical dealloying process.

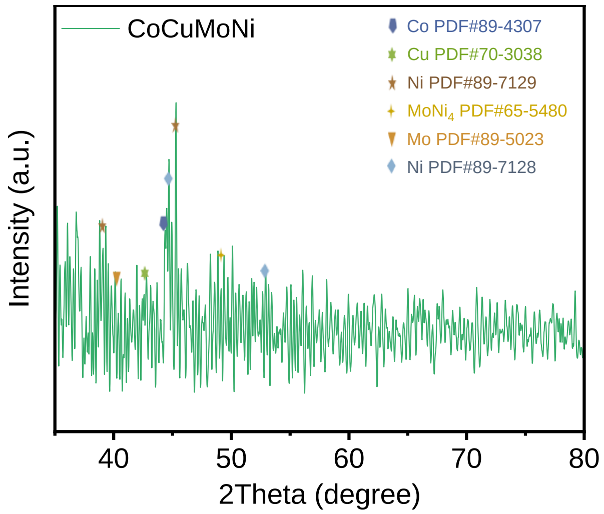

Figure S2. XRD spectra of CoCuMoNi.

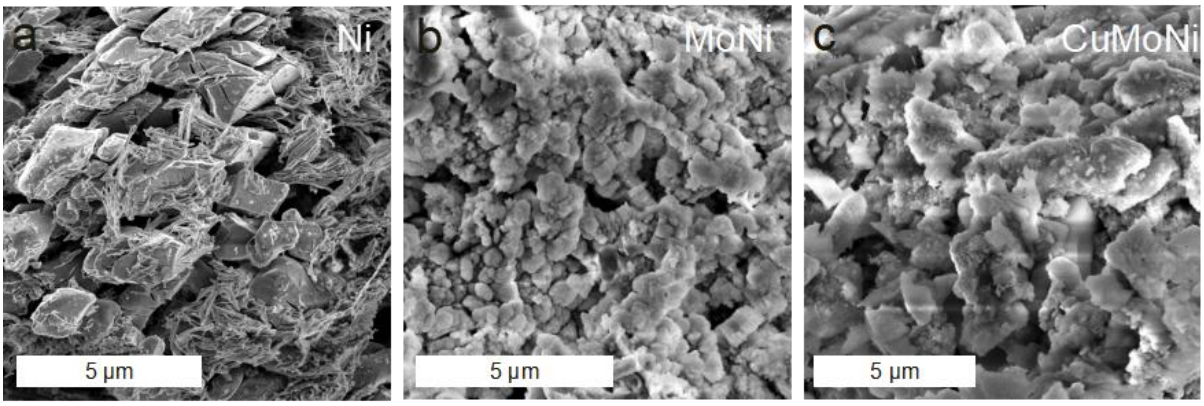

Figure S3. SEM images of (a) Ni, (b) MoNi, and (c) CuMoNi.

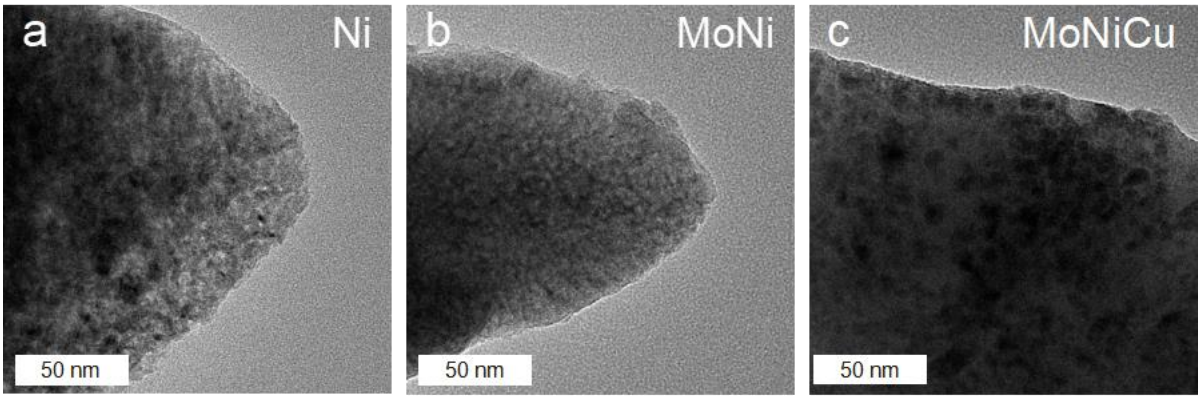

Figure S4. TEM images of (a) Ni, (b) MoNi, and (c) CuMoNi.

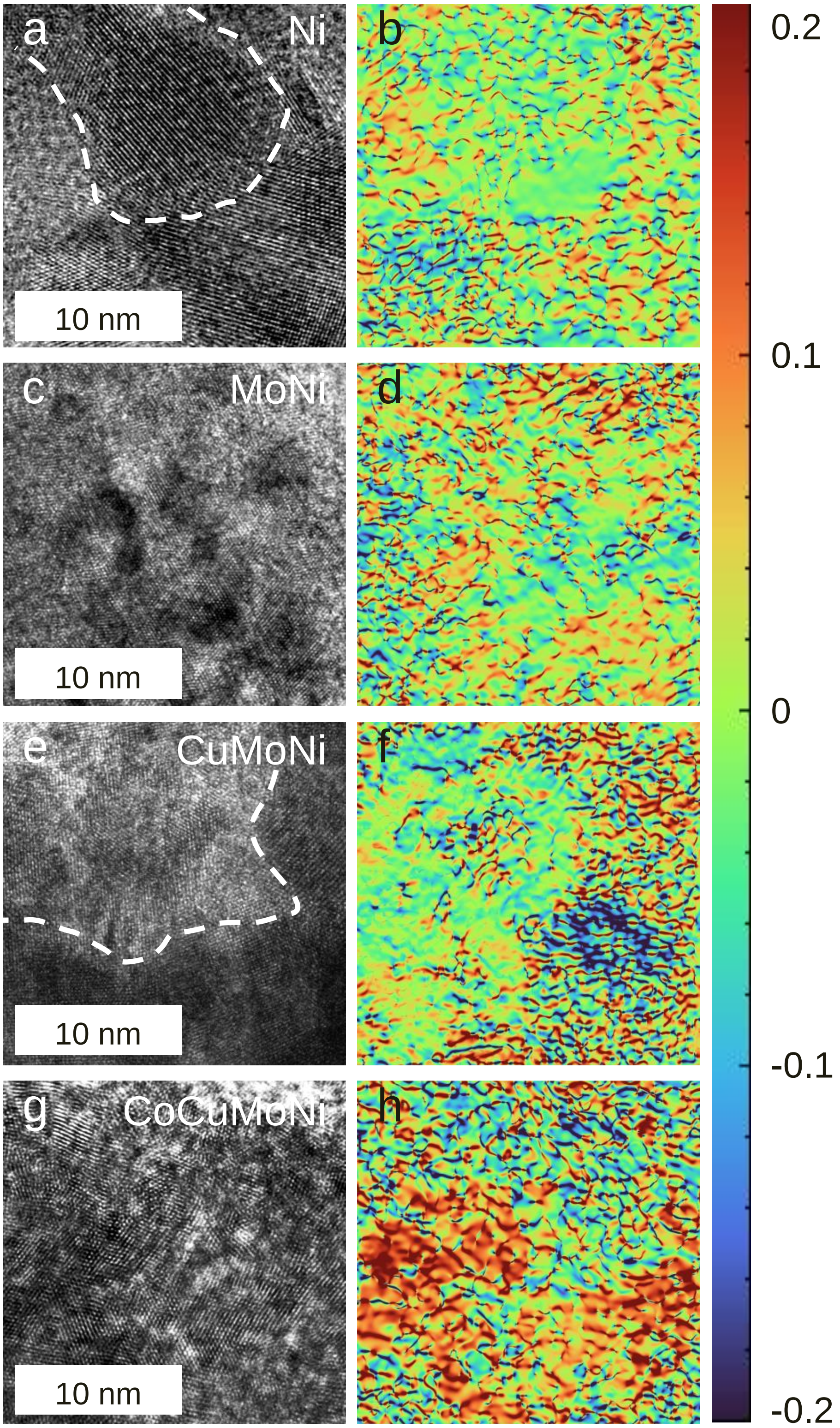

Figure S5. TEM images and corresponding strain distributions along E_{xy}. (a, b) Ni, (c, d) MoNi (e, f) CuMoNi and (g, h) CoCuMoNi. Compressive strain is represented by a shift from green to dark blue, while tensile strain is indicated by a shift from bright yellow to red.

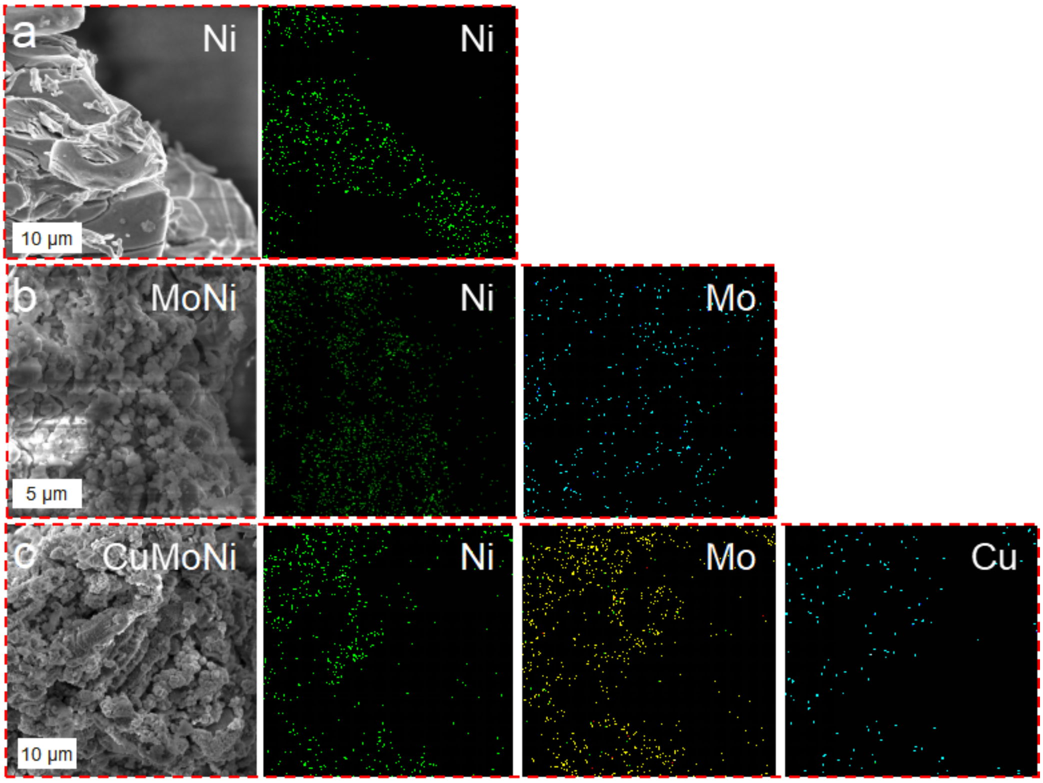

Figure S6. SEM images and corresponding EDS elemental mappings of (a) Ni, (b) MoNi, and (c) CuMoNi.

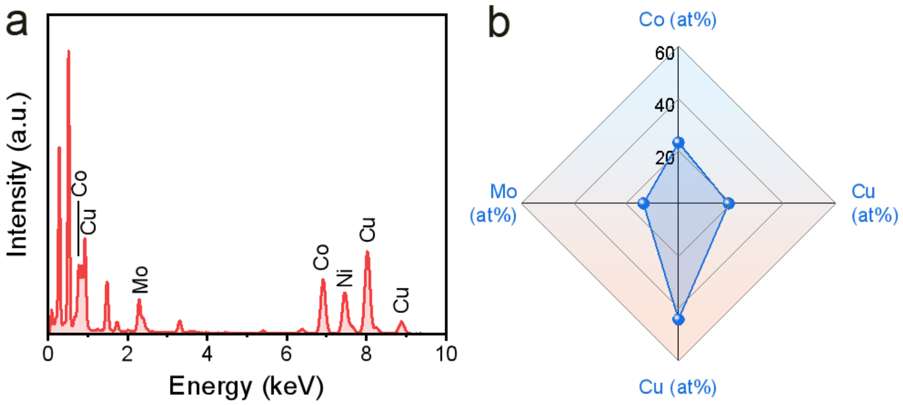

Figure S7. (a) EDS spectra and (b) the corresponding element content diagram of CoCuMoNi.

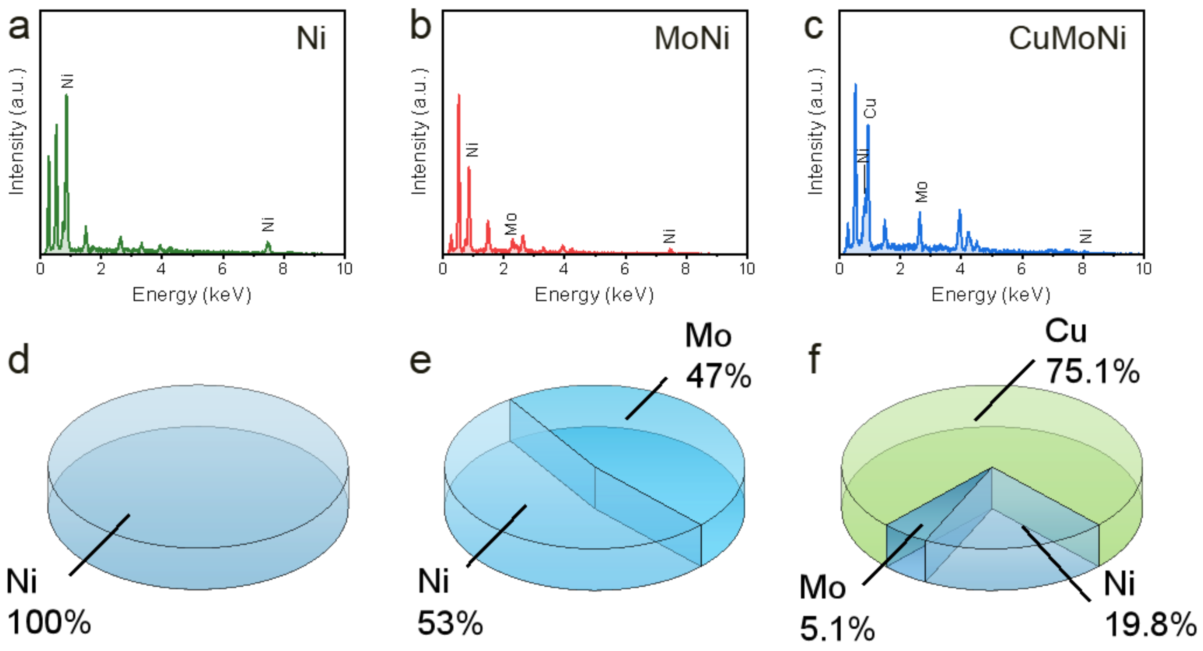

Figure S8. EDS spectra of (a) Ni, (b) MoNi, and (c) CuMoNi. The corresponding element content diagrams of (a) Ni, (b) MoNi, and (c) CuMoNi.

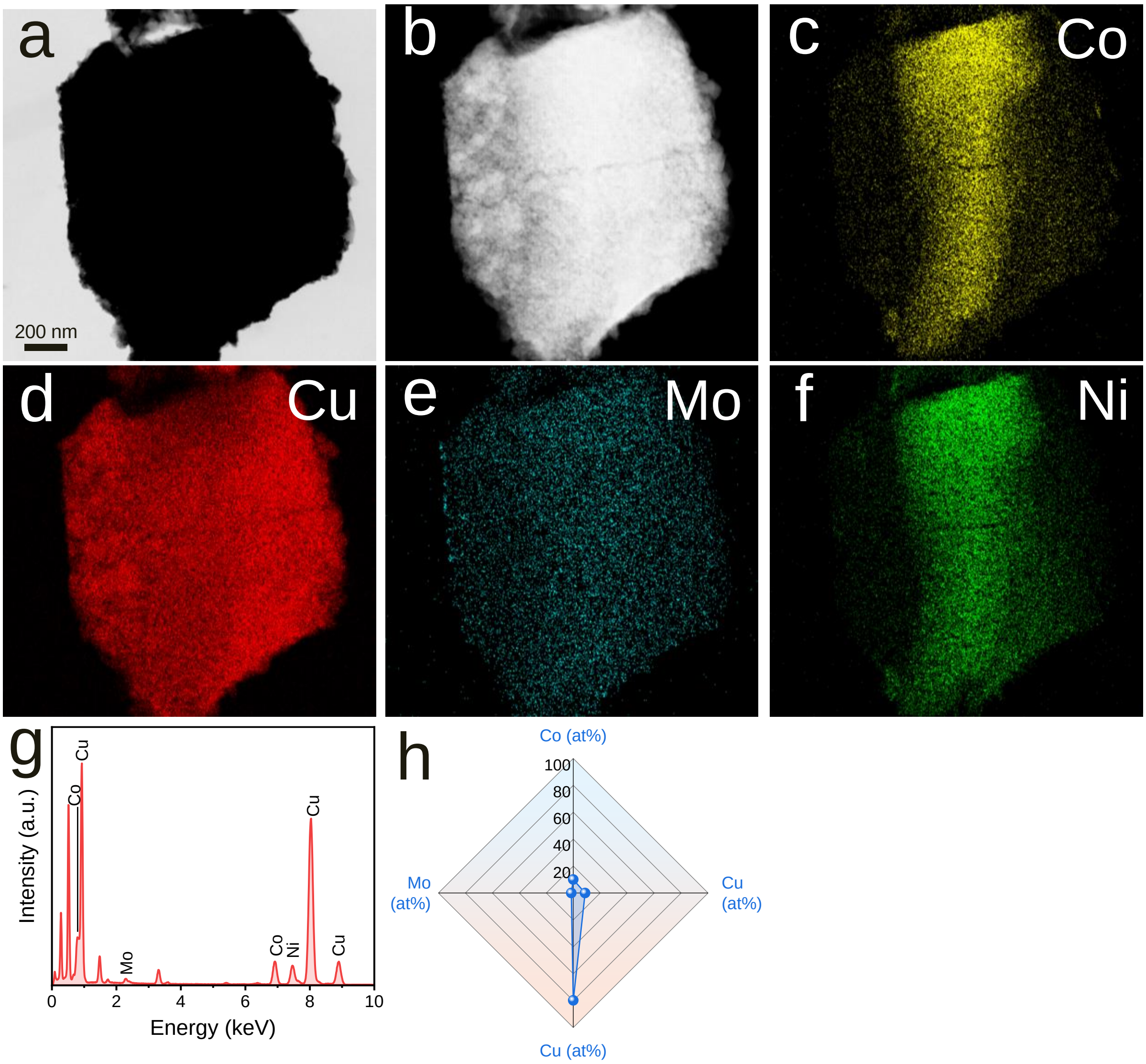

Figure S9. (a) TEM image, (b) STEM image, (c-f) the corresponding EDS element mappings, (g) EDS spectra, and (h) the corresponding element content diagram of CoCuMoNi.

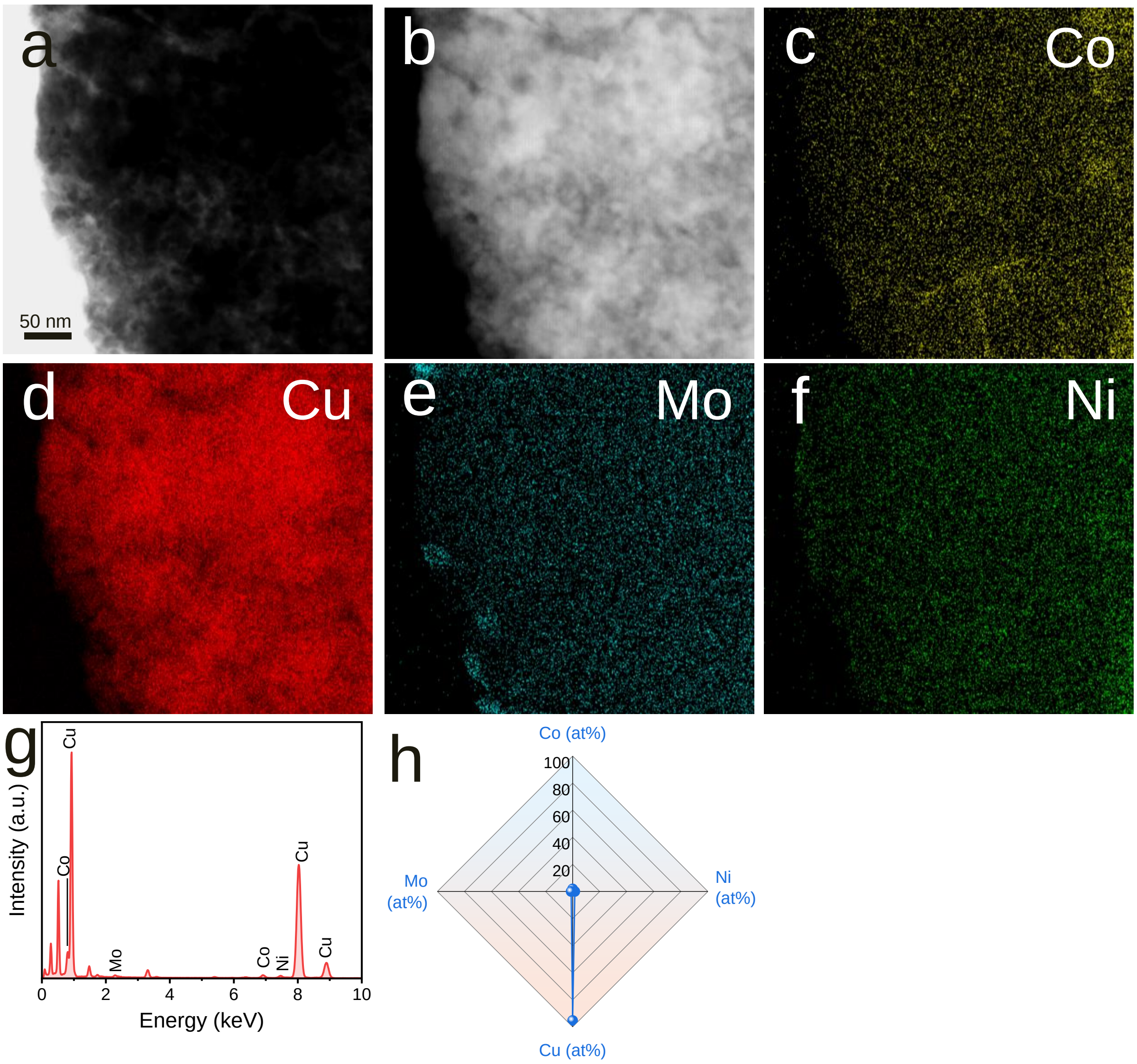

Figure S10. (a) TEM image, (b) STEM image, (c-f) the corresponding EDS element mappings, (g) EDS spectra, and (h) the corresponding element content diagram of CoCuMoNi.

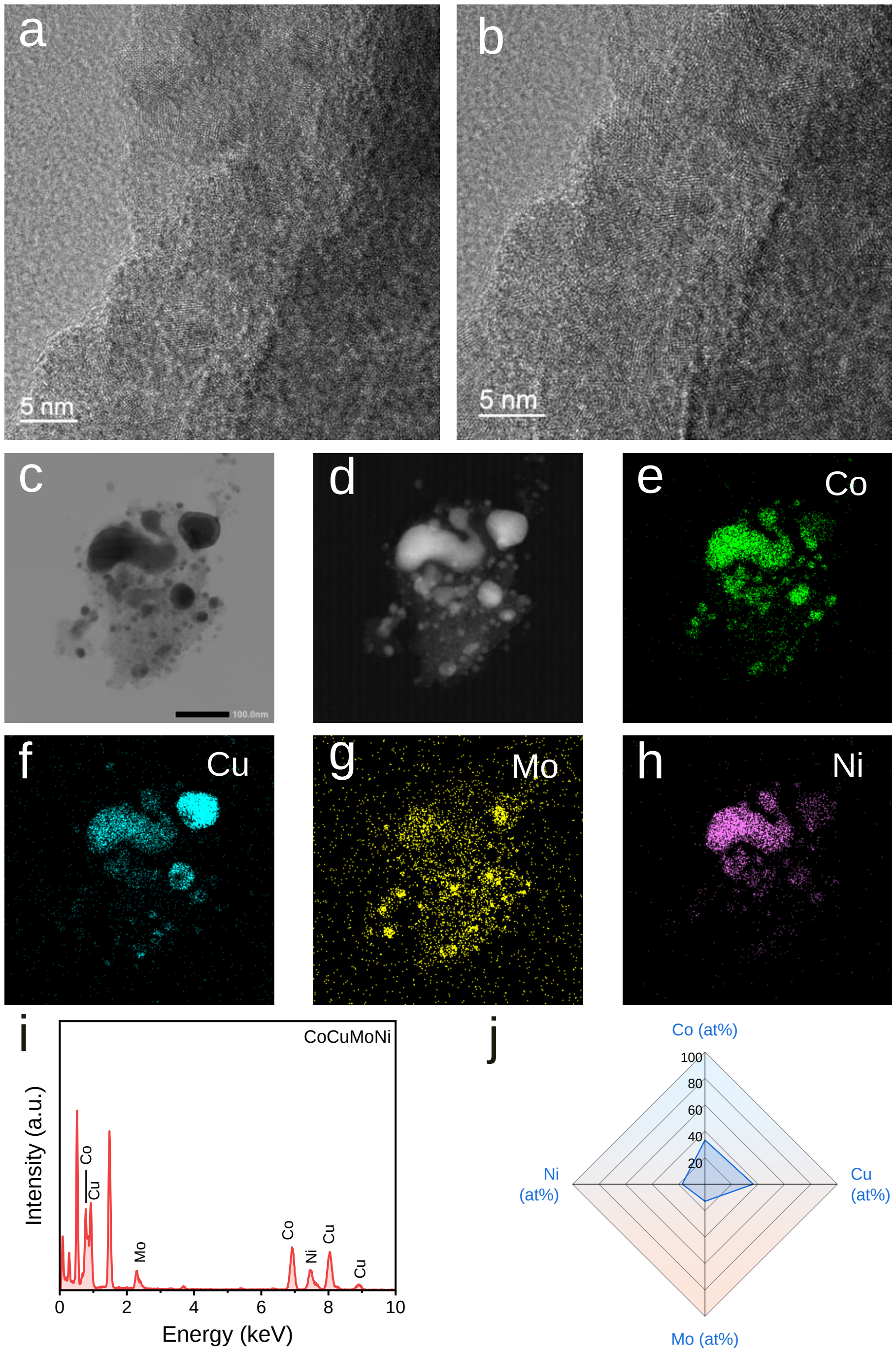

Figure S11. (a-c) TEM images, (d) STEM image, (e-h) the corresponding EDS element mappings, (i) EDS spectra, and (j) the corresponding element content diagram of CoCuMoNi.

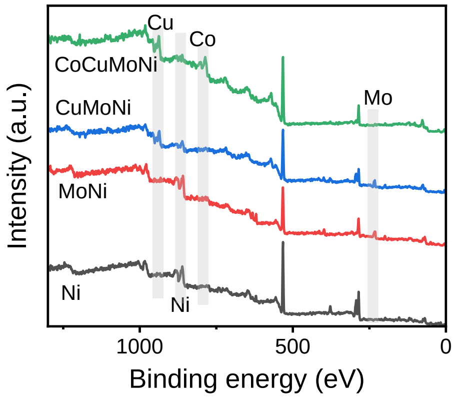

Figure S12. XPS survey spectra of Ni, MoNi, CuMoNi, and CoCuMoNi.

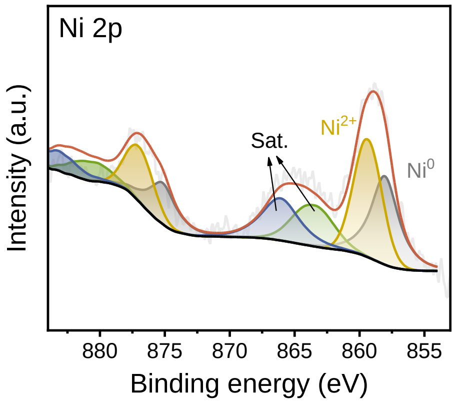

Figure S13. High-resolution XPS result of Ni.

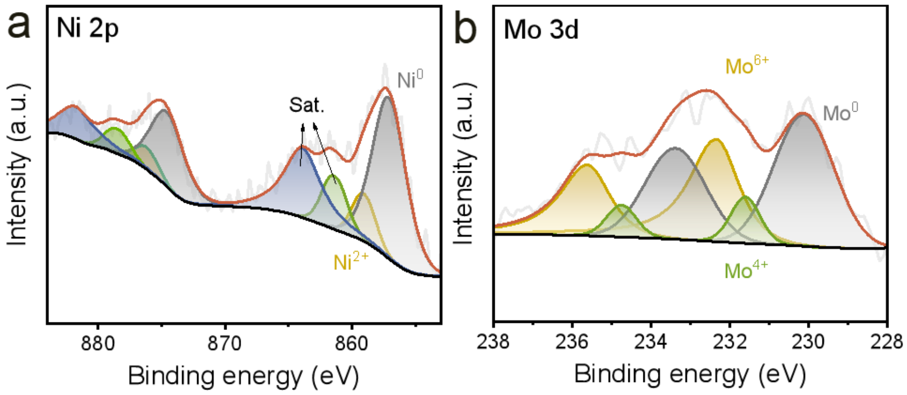

Figure S14. (a, b) High-resolution XPS results of MoNi.

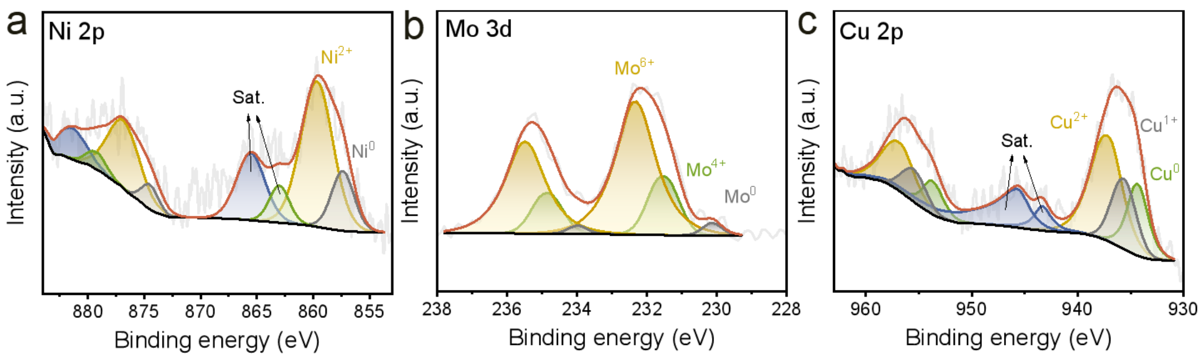

Figure S15. (a-c) High-resolution XPS results of CuMoNi.

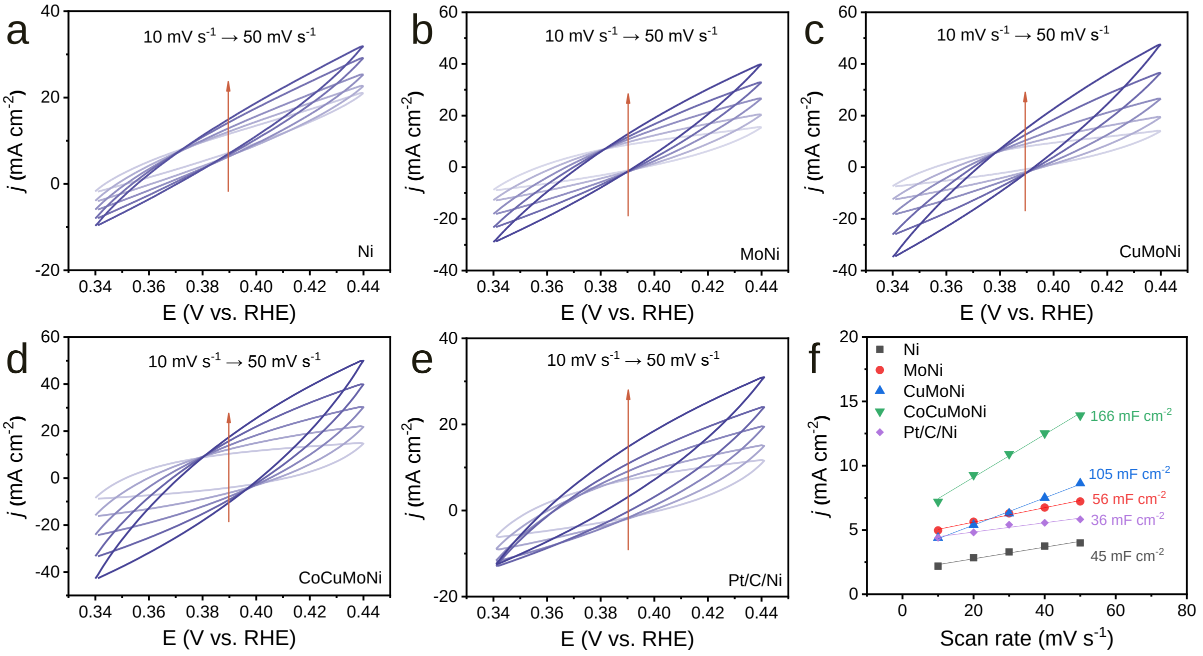

Figure S16. CV curves of (a) Ni, (b) MoNi, (c) CuMoNi, (d) CoCuMoNi and (e) Pt/C/Ni. (f) Capacitive current density versus scan rate for Ni, MoNi, CuMoNi, CoCuMoNi, and Pt/C/Ni.

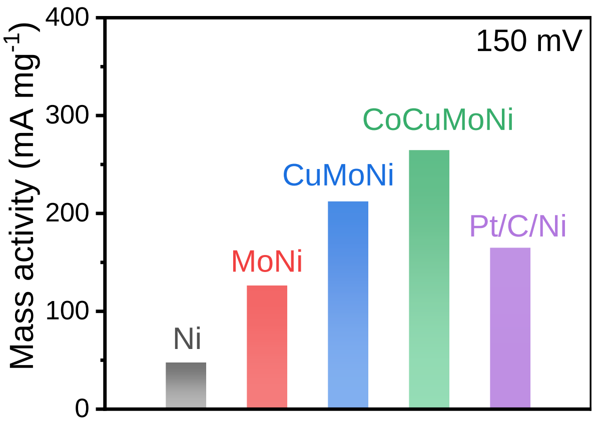

Figure S17. HER mass activities of Ni, MoNi, CuMoNi, CoCuMoNi, and Pt/C/Ni.

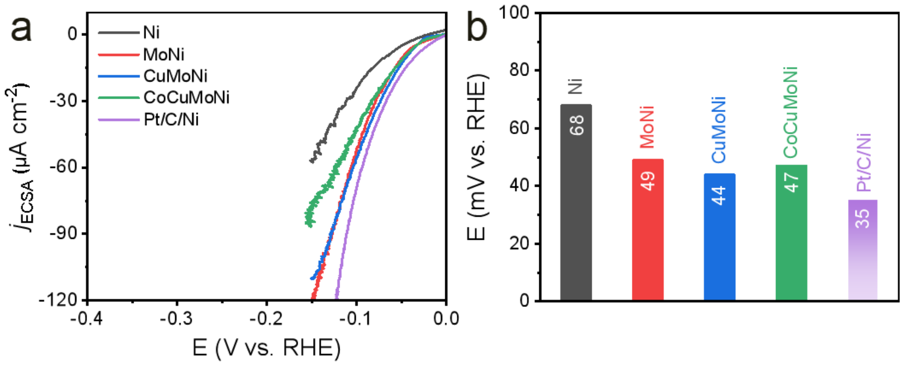

Figure S18. (a) The polarization curves normalized by ECSA and (b) corresponding overpotentials at 0.01 mA cm^{-2} of Ni, MoNi, CuMoNi, CoCuMoNi, and Pt/C/Ni toward HER.

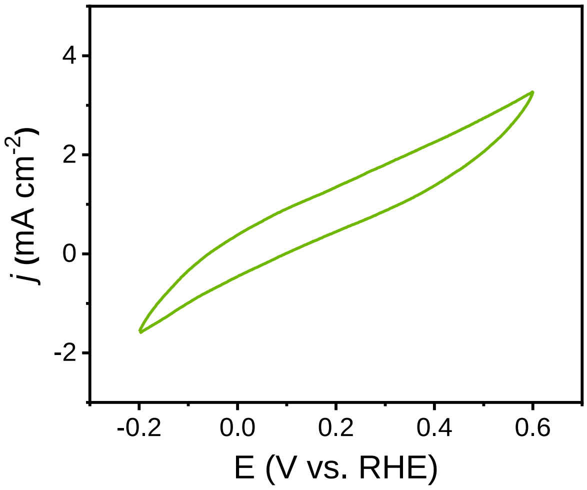

Figure S19. CV curve of CoCuMoNi sample in -0.2-6 V (vs. RHE) potential interval in 1 M PBS solution.

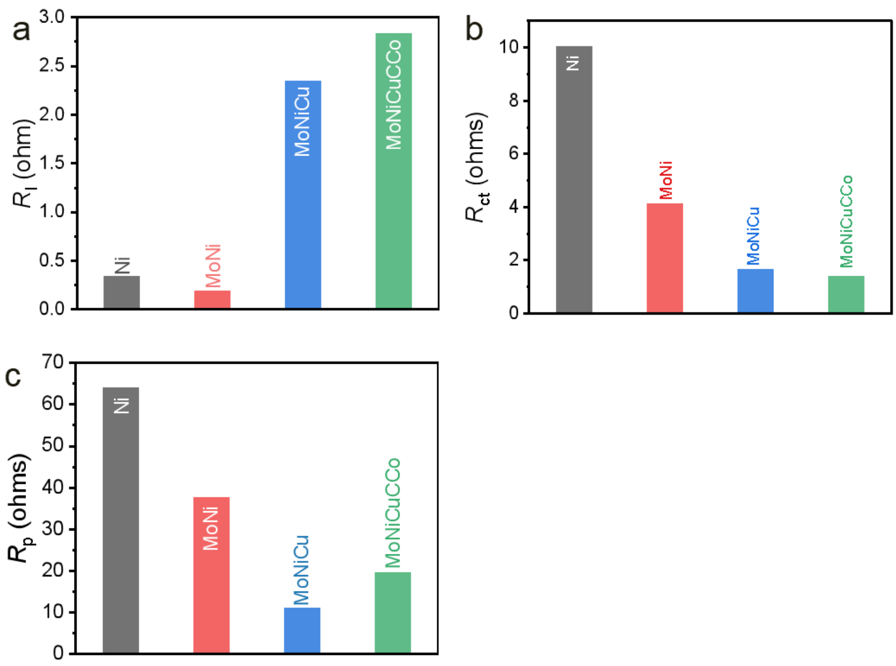

Figure S20 Comparison of (a) R_I, (b) R_{ct}, and (c) Rp for Ni, MoNi, CuMoNi, and CoCuMoNi electrodes according to EIS analysis.

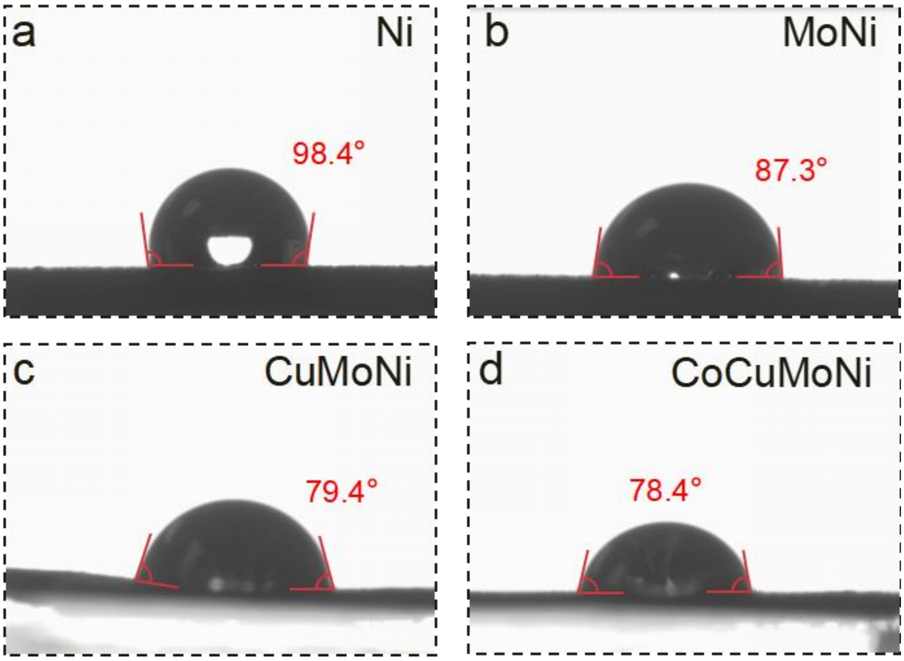

Figure S21. Contact angles of (a) Ni, (b) MoNi, (c) CuMoNi, and (d) CoCuMoNi.

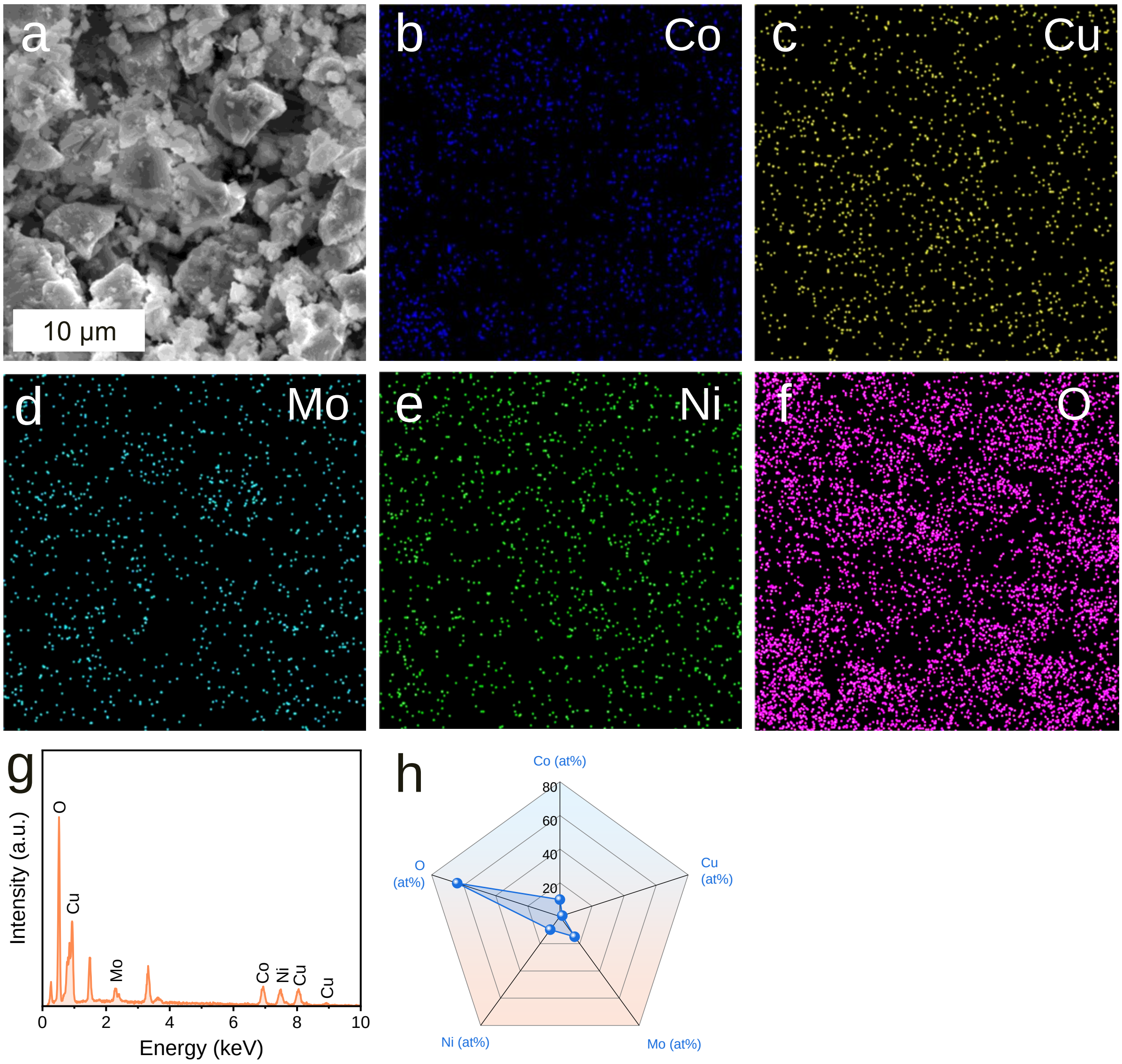

Figure S22. (a) SEM image, (b-f) the corresponding EDS element mappings, (g) EDS spectra, and (h) the corresponding element content diagram of CoCuMoNi after the stability test for HER.

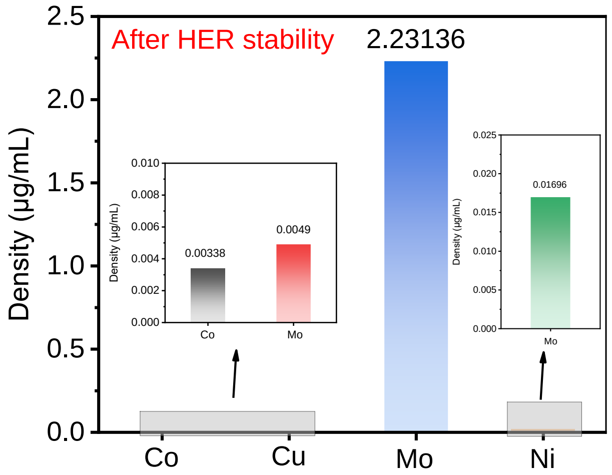

Figure S23. The ICP spectrum of the electrolyte after the HER stability test.

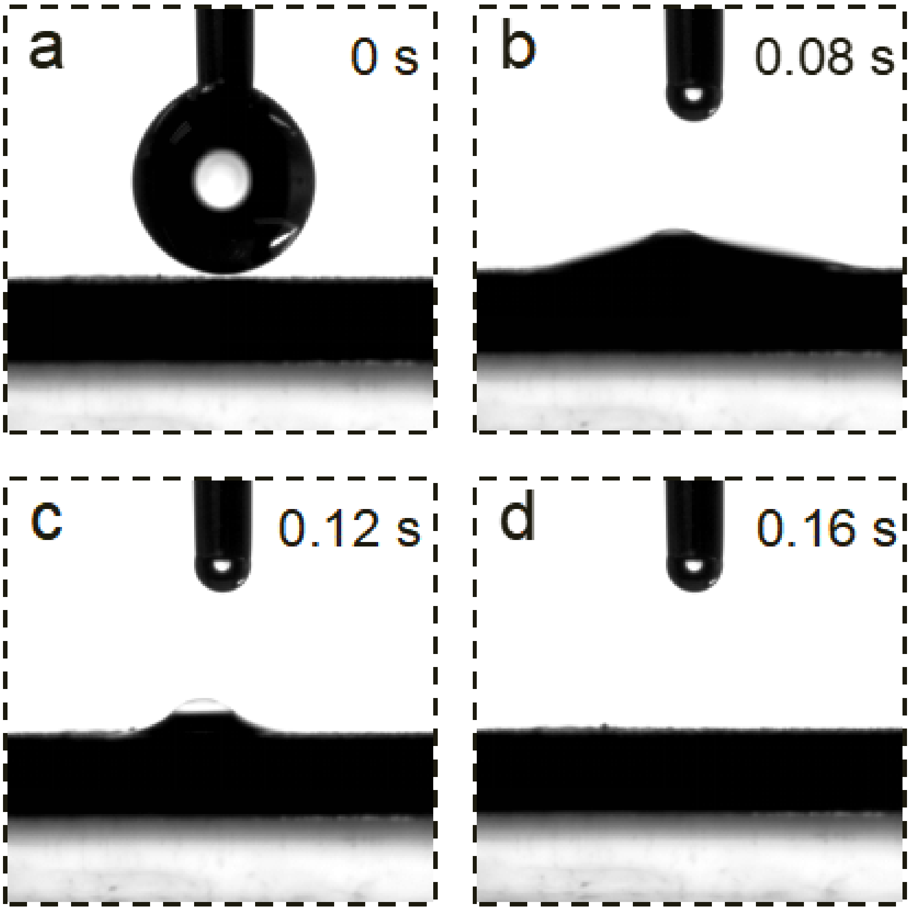

Figure S24. Contact angles of CoCuMoNi after stability test for HER.

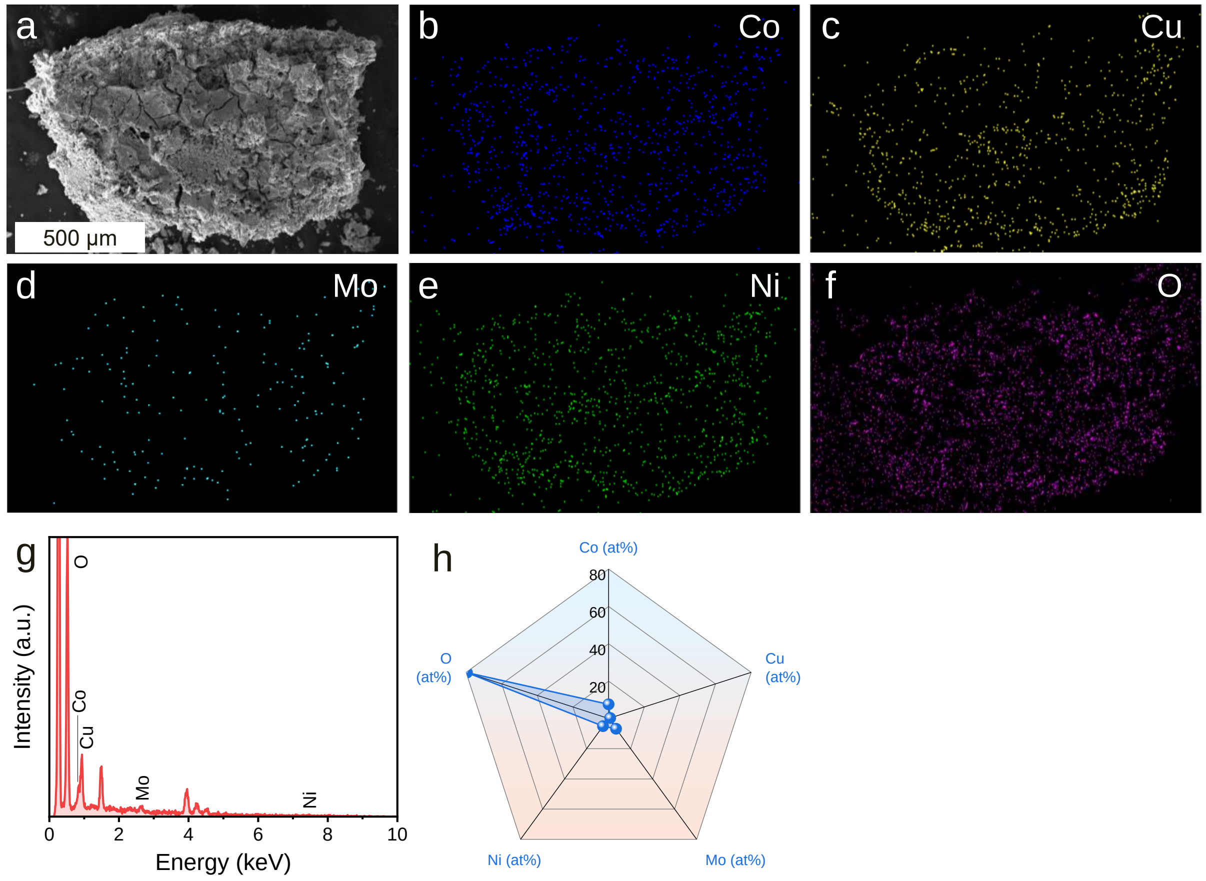

Figure S25. (a) SEM image, (b-f) the corresponding EDS element mappings, (g) EDS spectra, and (h) the corresponding element content diagram of CoCuMoNi after the stability test for OER.

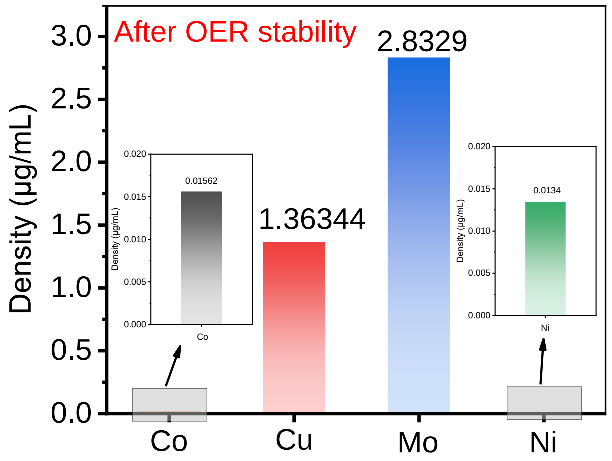

Figure S26. The ICP spectrum of the electrolyte after the OER stability test.

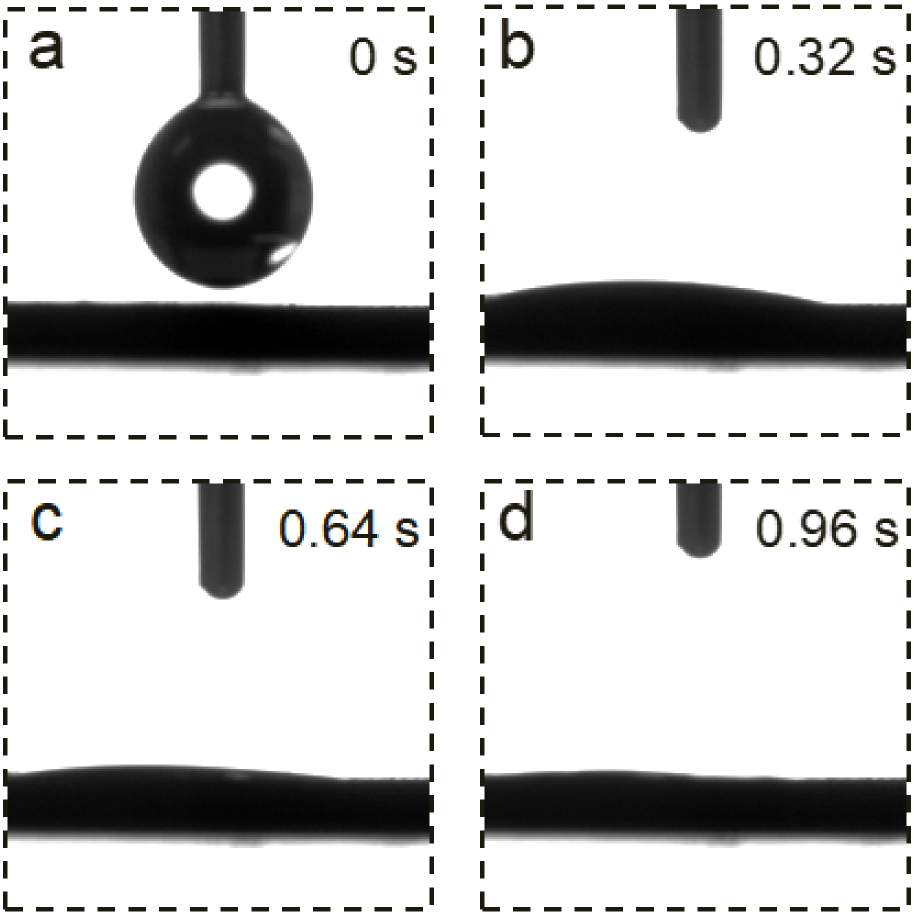

Figure S27. Contact angles of CoCuMoNi after stability test for OER.

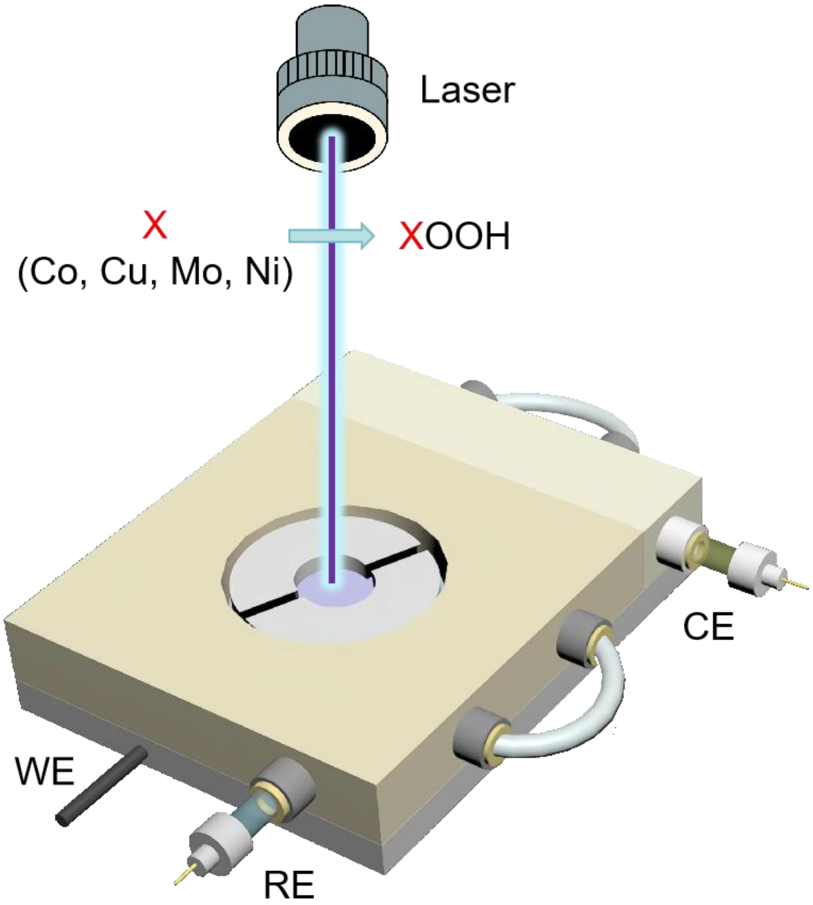

Figure S28. Schematic illustration of *in-situ* electrochemical Raman spectra of phase transformation of CoCuMoNi during OER stability test process.

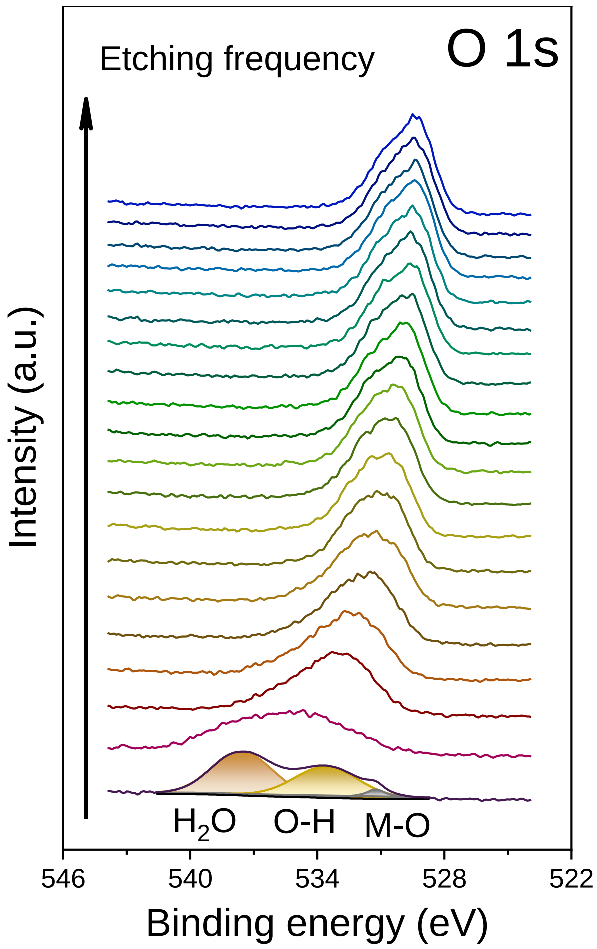

Figure S29. XPS spectra of O 1s of CoCuMoNi after stability test for OER.

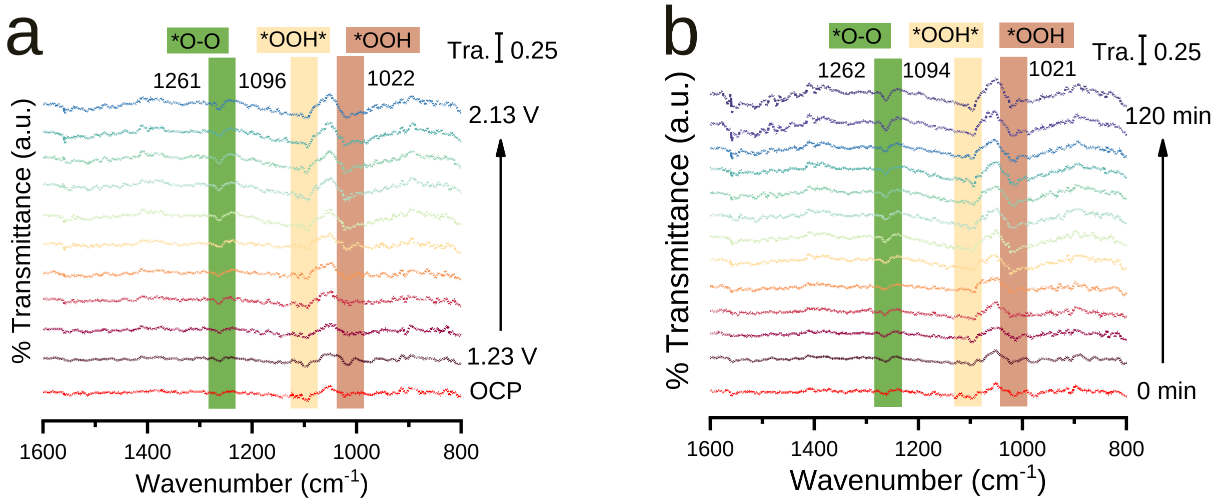

Figure S30. FT-IR measurements were conducted on the CoCuMoNi catalyst (a) under different applied potentials, and (b) during the 120-min OER stability test, respectively.

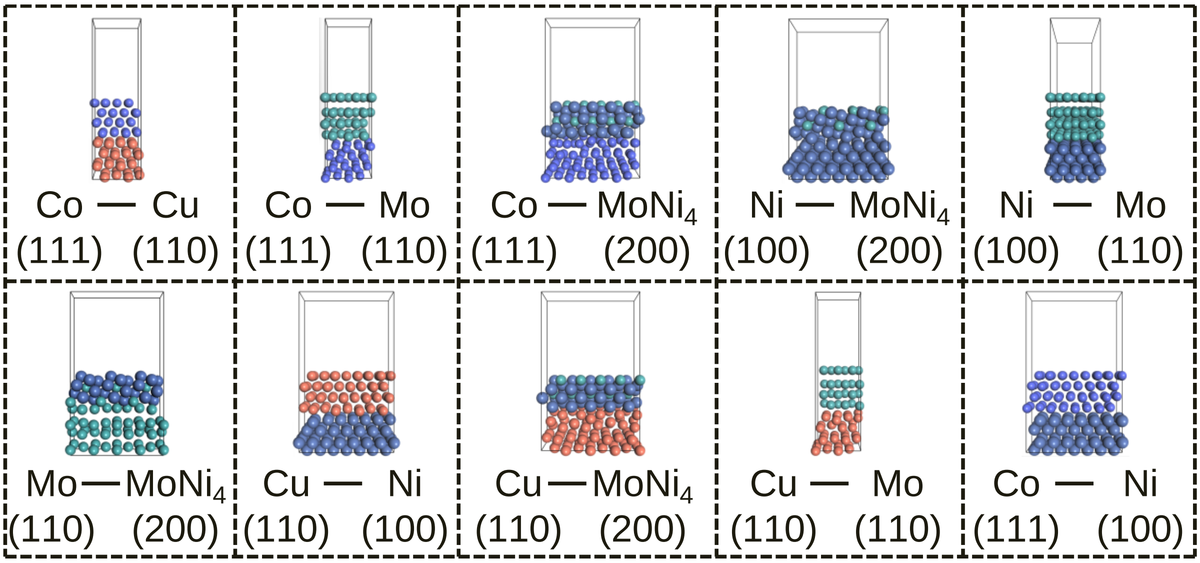

Figure S31. Three-dimensional models of different heterojunction structures.

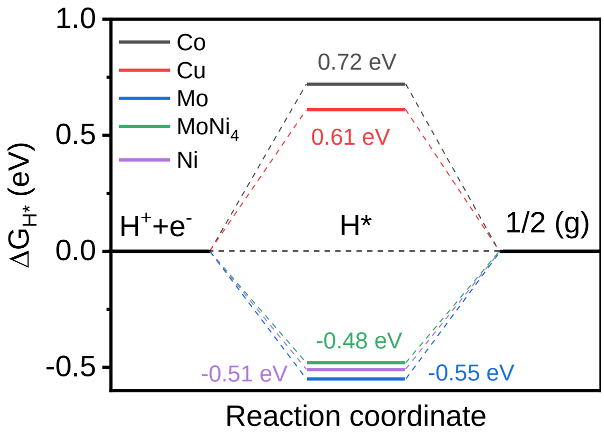

Figure S32. Free energy profiles for HER of monomer.

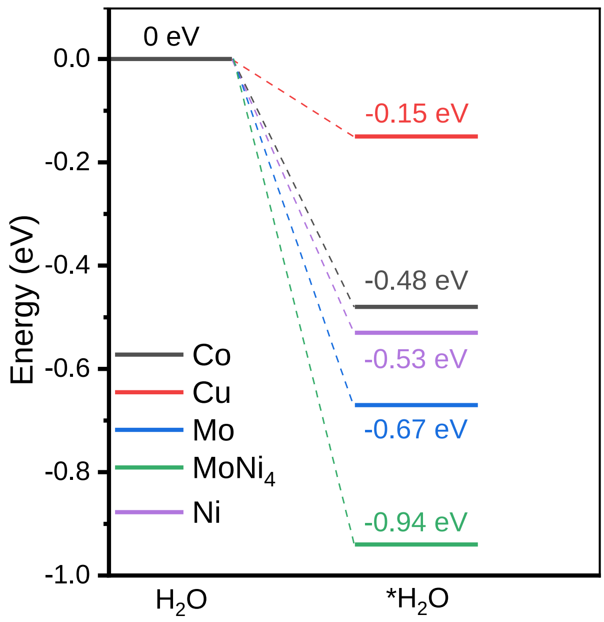

Figure S33. $E_{ads}(H_2O)$ of monomer.

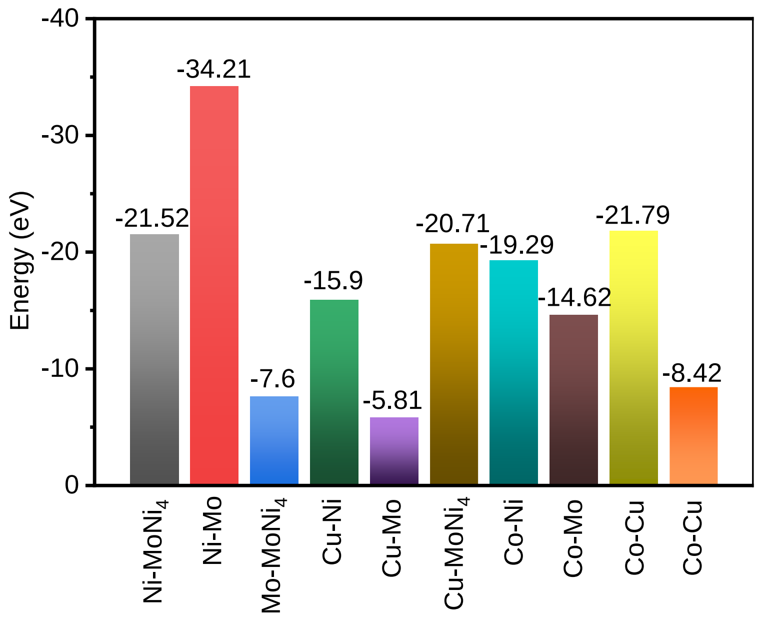

Figure S34. The formation energies of different heterojunctions.

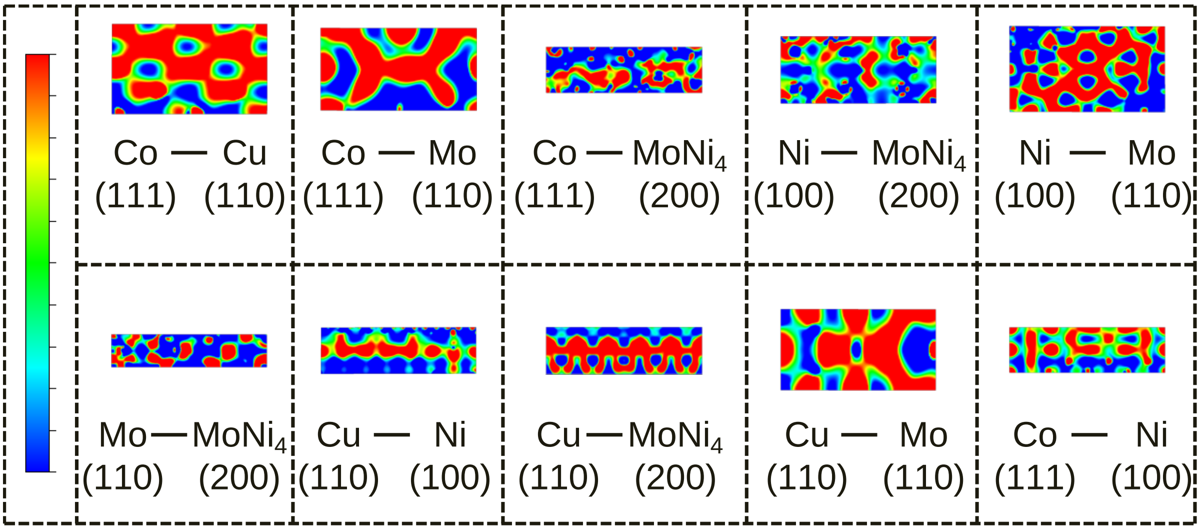

Figure S35. Differential charge density cross-sectional diagrams at different heterojunction interfaces. The darker red and blue colors correspond to a stronger tendency for electron depletion and accumulation, respectively.

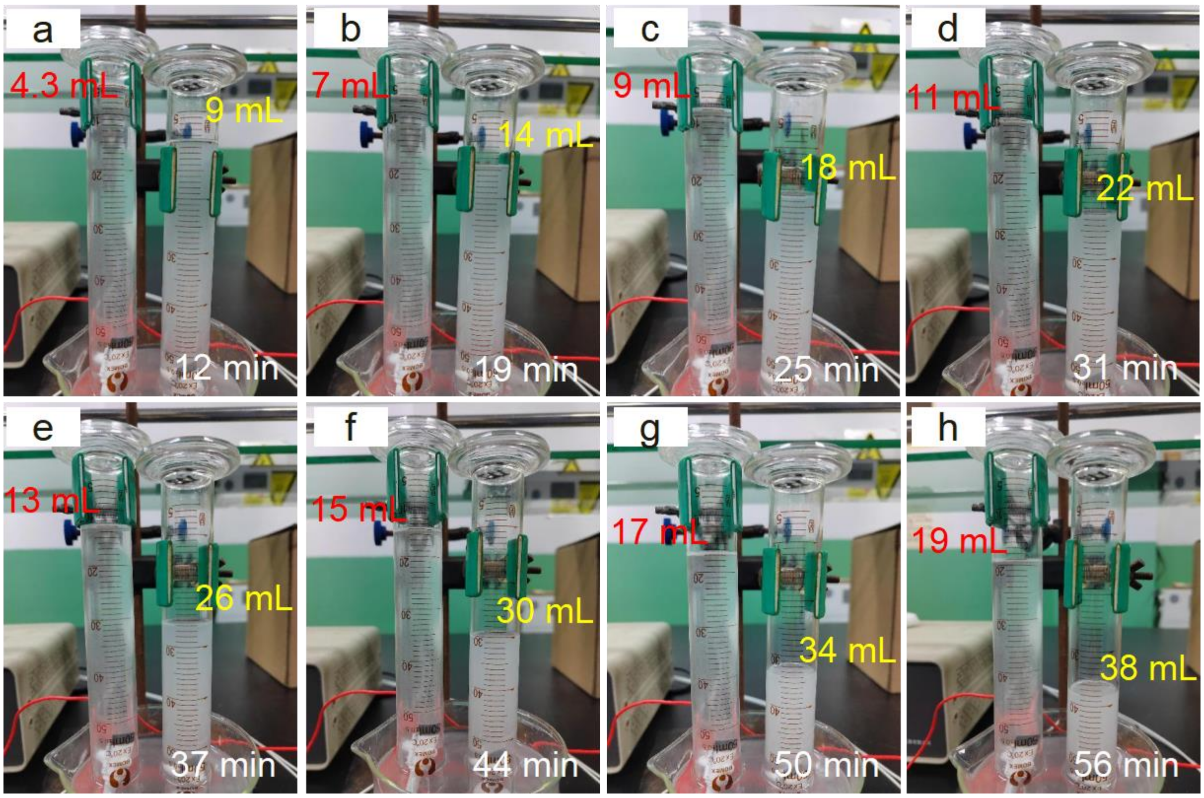

Figure S36. (a-h) Digital photograph of hydrogen and oxygen gas generated at 0, 12, 19, 25, 31, 37, 44, 50 and 56 min, respectively.

Table S1. HER activities of the as-synthesized CoCuMoNi electrocatalyst compared with reported related electrocatalysts (at 10 mA cm^{-2} in 1 M KOH solution).

Electrocatalysts		η (mV)	Tafel (mV dec^{-1})	Reference
**CoCuMoNi**		**14**	**10**	**This work**
Fe-based	FeS_2	96	78	[1]
	B-Fe_7S_8/FeS_2	113	57	[2]
	Fe–CoP/Ti	78	75	[3]
	FeS_2 NSs	186	123	[4]
Co-based	c-$CoSe_2$/CC	190	85	[5]
	Co/CoP-5	253	73.8	[6]
	Co_9S_8@NOSCb	320	105	[7]
	CoO_x@CN	232	115	[8]
	HNDCM-Co/CoP	138	64	[9]
	Cr-Co_xP	100	75	[10]
	Fe@Co_9S_8	44	66	[11]
Ni-based	Ni_3N@CQDs	69	108	[12]
	Ni/Ni_xP_y	130	58.5	[13]
	Ni_3S_2/MoS_2	110	55	[14]
	NF-NiS_2	67	63	[15]
	Co-Ni_3S_2@CNT/GNF	155	138	[16]
	Mo-Ni_3S_2	212	98	[17]
	N-Ni_3S_2	155	113	[18]
	m-$NiTP_yP$	138	83	[19]
Multiple metal-based	FeCoNi	64	125	[20]
	$NiFeO_x$@NiCu	70	68	[21]
	NiZn-CoO	53	47	[22]
	Ni_4Mo/MoO_x/Cu	16	64	[23]
	NiFeSe	50	49	[24]
	NiCoWS	70	112	[25]
	H-Fe-CoMoS	137	98	[26]
	$Fe_{0.54}Co_{0.46}S_{0.92}$/CNTs/C	70	64	[27]
	NiFe@MoS_2	67	42	[28]
Noble-metal-based	β-$Ni(OH)_2$/Pt	92	51	[29]
	Sr_2RuO_4	61	50	[30]
	IrP_2@NC	28	43	[31]
	CoNiPt NFs	25	48	[32]
	$Cu_{2-x}S$@Ru	82	29	[33]
	Ru/NC	24	24	[34]
	Mo-$RuCoO_x$	41	42	[35]

Table S2. TOF of the as-synthesized CoCuMoNi electrocatalyst for HER, compared with reported related electrocatalysts (in 1 M KOH solution).

Electrocatalysts	TOF (s^{-1})	Overpotential (V)	Reference
**CoCuMoNi**	**3.61**	**0.025**	**This work**
	**16.39**	**0.05**	
	**34.97**	**0.075**	
	**57.79**	**0.1**	
	**91.43**	**0.13**	
$Ru@C_2N$	0.75	0.025	[36]
PtPdRhRuCu	4.5	0.05	[37]
W-ACs	0.12	0.05	[38]
FeCoNiCuPtIr	0.311	0.05	[39]
Rh_{15}/NSC	0.143	0.075	[40]
Ni_2P	0.015	0.1	[41]
Ni_4Mo/GNS	0.11	0.1	[42]
FeCoNiRu-450	0.046	0.1	[43]
B-Os aerogels	1.17	0.1	[44]
W-$NiS_{0.5}Se_{0.5}$	0.21	0.13	[45]

Table S3. OER activities of the as-synthesized CoCuMoNi electrocatalyst compared with reported related electrocatalysts (at 10 mA cm^{-2} in 1 M KOH solution).

Electrocatalysts		η (mV)	Tafel (mV dec^{-1})	Reference
	**CoCuMoNi**	**211**	**45**	**This work**
Fe-based	Fe(III)-Bir	240	33	[46]
	NiFe LDH	300	40	[47]
	FeCo LDH	331	85	[48]
	P/Fe-N-C	304	65	[49]
	MIL-53(Fe)-2OH	215	45	[50]
Co-based	$CoMoO_4$	312	56	[51]
	Co_3O_4	400	49	[52]
	CoS	361	64	[53]
	CoP/rGO-400	340	66	[54]
	Co_3O_4/rm-GO	310	67	[55]
	N-CG-CoO	340	71	[56]
	CoCo-NS	353	45	[57]
	CoO_x	423	42	[58]
	Co–P FILM	345	47	[59]
	Co–P/NC	354	52	[60]
	Co_3O_4 NCs	350	101	[61]
Ni-based	m-$NiTP_yP$	267	33	[19]
	MoS_2/NiS_2	278	91	[62]
	NiS	335	89	[63]
	Ni_3N nanosheets	350	85	[64]
	Ni@NC	370	45	[65]
	NiS-$Ni(OH)_2$@aMoS_{2+x}	417	97	[66]
Multiple metal-based	NiFe@MoS_2	201	48	[28]
	Mo-$RuCoO_x$	156	69	[35]
	$Fe_{20}Co_{20}Ni_{20}Mo_{20}Al_{20}$	223	39	[67]
	AlNiCoRuMo	245	54	[68]
	CoCrFeNiMo	220	30	[69]
	$FeNiCoCrMnS_2$	199	39	[70]
	$(CrFeCoNi)_{97}O_3$	196	29	[71]
	FeCoNiCrMn	229	40	[72]
	FeCoNiPB	235	53	[73]
	FeCoNiMnCu	280	59	[74]
	$Fe_{0.5}CoNiCuZn_{0.8}$	340	48	[75]
Noble-metal-based	NiFeRu-LDH	290	32.4	[76]
	Ir/C-Pt/C couple	370	58.6	[76]
	IrO_2	338	47	[57]
	Ir/C	320	54	[77]

Table S4. TOF of the as-synthesized CoCuMoNi electrocatalyst for OER, compared with reported related electrocatalysts (in 1 M KOH solution).

Electrocatalysts	TOF (s^{-1})	Overpotential (V)	Reference
**CoCuMoNi**	**0.61**	**1.43**	**This work**
	**2.18**	**1.48**	
	**3.15**	**1.5**	
	**5.23**	**1.53**	
	**6.16**	**1.54**	
	**7.06**	**1.55**	
	**8.06**	**1.56**	
	**13.41**	**1.6**	
KC-MLH/NF-12	0.09	1.43	[78]
	0.37	1.48	
Ru/Co_3O_{4-x}	0.056	1.5	[79]
FeCoNiMo HEA	0.051	1.53	[80]
S/N-CMF@Fe_xNi_{1-x}-MOF	0.124	1.53	[81]
m-NiTPyP/CNTs	1.16	1.53	[82]
MIL-53(Fe)-2OH	1.44	1.53	[50]
Rh-RuO_2/G	1.74	1.53	[83]
	2.39	1.53	
Co@NPC-Tfu	3.21	1.53	[84]
NiMoN/NiFe LDH	3.39	1.53	[85]
$Co_{0.5}Fe_{0.5}$-LDH	0.128	1.54	[86]
$Ir_{0.1}Ta_{0.9}O_{2.45}$	2.3	1.55	[87]
FeCoNiRu-450	0.084	1.56	[43]
Mo-$RuCoO_x$	0.176	1.6	[35]
Ir-MnO2(160)-CC	0.321	1.6	[88]

Table S5. Water splitting activities of the as-synthesized CoCuMoNi electrocatalyst, compared with reported related electrocatalysts (in 1 M KOH solution).

Electrocatalysts	η (V@100 mA cm^{-2})	Reference
CoCuMoNi	**1.559**	**This work**
Raynel Ni‖Ni mesh	1.760	[89]
Co/CoMoN/NF	1.56	[90]
$Ce_{0.2}$-CoP/Ni_3P@NF‖$Ce_{0.2}$-FeP_x/Ni_3P@NF	1.561	[91]
CoP_2-Mo_4P_3/NF	1.59	[92]
Ni_5Co_3Mo-OH	1.6	[93]
Ni_2P-Ni_3S_2 HNAs/NF	1.62	[94]
FL-$CoFe_2O_4$/NF‖P-NiMoP/NF	1.63	[95]
NiFe/Y_2O_3	1.64	[96]
NiCoP@NiMn LDH/NF	1.642	[97]
CoMoNiP	1.65	[98]
Ni_3S_2/VO_2	1.65	[99]
CoP@NiFe-OH/SPNF	1.68	[100]
Cu@NiFe LDH	1.69	[101]
Cu@GDY-Co	1.7	[102]
Fe-Ni_3S_2/NF	1.7	[103]
Mo-NiP_x/NiS_y	1.7	[104]
$(Ni_{0.33}Fe_{0.67})_2P$	1.7	[105]
Cr-CoP/CP	1.73	[106]
W,Mo-NiCoP/NF	1.74	[107]
Mn-Ni_3Se_2@NF	1.776	[108]
NiCoP@NF-100	1.8	[109]

References

[1] R. Miao, B. Dutta, S. Sahoo, J. He, W. Zhong, S. A. Cetegen, T. Jiang, S. P. Alpay, S. L. Suib, *J. Am. Chem. Soc.* **2017**, *139*, 13604.

[2] J. Wu, Q. Zhang, K. Shen, R. Zhao, W. Zhong, C. Yang, H. Xiang, X. Li, N. Yang, *Adv. Funct. Mater.* **2022**, *32*, 2107802.

[3] C. Tang, R. Zhang, W. Lu, L. He, X. Jiang, A. M. Asiri, X. Sun, *Adv. Mater.* **2017**, *29*, 1602441.

[4] Y. Li, J. Yin, L. An, M. Lu, K. Sun, Y. Q. Zhao, D. Gao, F. Cheng, P. Xi, *Small* **2018**, *14*, 1801070.

[5] P. Chen, K. Xu, S. Tao, T. Zhou, Y. Tong, H. Ding, L. Zhang, W. Chu, C. Wu, Y. Xie, *Adv. Mater.* **2016**, *28*, 7527.

[6] Z. H. Xue, H. Su, Q. Y. Yu, B. Zhang, H. H. Wang, X. H. Li, J. S. Chen, *Adv. Energy Mater.* **2017**, *7*, 1602355.

[7] S. Huang, Y. Meng, S. He, A. Goswami, Q. Wu, J. Li, S. Tong, T. Asefa, M. Wu, *Adv. Funct. Mater.* **2017**, *27*, 1606585.

[8] H. Jin, J. Wang, D. Su, Z. Wei, Z. Pang, Y. Wang, *J. Am. Chem. Soc.* **2015**, *137*, 2688.

[9] H. Wang, S. Min, Q. Wang, D. Li, G. Casillas, C. Ma, Y. Li, Z. Liu, L.-J. Li, J. Yuan, *ACS Nano* **2017**, *11*, 4358.

[10] Y. Song, M. Sun, S. Zhang, X. Zhang, P. Yi, J. Liu, B. Huang, M. Huang, L. Zhang, *Adv. Funct. Mater.* **2023**, *33*, 2214081.

[11] B. Tian, L. G. Sun, D. R. Ho, *Adv. Funct. Mater.* **2023**, *33*, 2210298.

[12] M. Zhou, Q. Weng, Z. I. Popov, Y. Yang, L. Y. Antipina, P. B. Sorokin, X. Wang, Y. Bando, D. Golberg, *ACS Nano* **2018**, *12*, 4148.

[13] G. F. Chen, T. Y. Ma, Z. Q. Liu, N. Li, Y. Z. Su, K. Davey, S. Z. Qiao, *Adv. Funct. Mater.* **2016**, *26*, 3314.

[14] J. Zhang, T. Wang, D. Pohl, B. Rellinghaus, R. Dong, S. Liu, X. Zhuang, X. Feng, *Angew. Chem.* **2016**, *128*, 6814.

[15] Q. Ma, C. Hu, K. Liu, S.-F. Hung, D. Ou, H. M. Chen, G. Fu, N. Zheng, *Nano Energy* **2017**, *41*, 148.

[16] F. Wang, Y. Zhu, W. Tian, X. Lv, H. Zhang, Z. Hu, Y. Zhang, J. Ji, W. Jiang, *J. Mater. Chem. A* **2018**, *6*, 10490.

[17] C. Wu, B. Liu, J. Wang, Y. Su, H. Yan, C. Ng, C. Li, J. Wei, *Appl. Surf. Sci.* **2018**, *441*, 1024.

[18] T. Kou, T. Smart, B. Yao, I. Chen, D. Thota, Y. Ping, Y. Li, *Adv. Energy Mater.* **2018**, *8*, 1703538.

[19] Y. Y. Zhang, S. T. Chen, Y. X. Zhang, R. J. Li, B. Zhao, T. Y. Peng, *Adv. Mater.* **2023**, *35*, 2210727.

[20] Q. Zhang, N. M. Bedford, J. Pan, X. Lu, R. Amal, *Adv. Energy Mater.* **2019**, *9*, 1901312.

[21] Y. Zhou, Z. Wang, Z. Pan, L. Liu, J. Xi, X. Luo, Y. Shen, *Adv. Mater.* **2019**, *31*, 1806769.

[22] T. Ling, T. Zhang, B. Ge, L. Han, L. Zheng, F. Lin, Z. Xu, W. B. Hu, X. W. Du, K. Davey, *Adv. Mater.* **2019**, *31*, 1807771.

[23] Y. An, X. Long, M. Ma, J. Hu, H. Lin, D. Zhou, Z. Xing, B. Huang, S. Yang, *Adv. Energy Mater.* **2019**, *9*, 1901454.

[24] G. Yilmaz, C. F. Tan, Y. F. Lim, G. W. Ho, *Adv. Energy Mater.* **2019**, *9*, 1802983.

[25] M. Ma, J. Xu, H. Wang, X. Zhang, S. Hu, W. Zhou, H. Liu, *Appl. Catal. B-Environ.* **2021**, *297*, 120455.

[26] Y. Guo, X. Zhou, J. Tang, S. Tanaka, Y. V. Kaneti, J. Na, B. Jiang, Y. Yamauchi, Y. Bando, Y. Sugahara, *Nano Energy* **2020**, *75*, 104913.

[27] W. Xiong, Z. Guo, H. Li, R. Zhao, X. Wang, *ACS Energy Lett.* **2017**, *2*, 2778.

[28] Z. Jiang, W. Zhou, C. Hu, X. Luo, W. Zeng, X. Gong, Y. Yang, T. Yu, W. Lei, C. Yuan, *Adv. Mater.* **2023**, *35*, 2300505.

[29] X. Yu, J. Zhao, L.-R. Zheng, Y. Tong, M. Zhang, G. Xu, C. Li, J. Ma, G. Shi, *ACS Energy Lett.* **2017**, *3*, 237.

[30] Y. Zhu, H. A. Tahini, Z. Hu, J. Dai, Y. Chen, H. Sun, W. Zhou, M. Liu, S. C. Smith, H. Wang, *Nat. Commun.* **2019**, *10*, 149.

[31] Z. Pu, J. Zhao, I. S. Amiinu, W. Li, M. Wang, D. He, S. Mu, *Energy Environ. Sci.* **2019**, *12*, 952.

[32] Y. D. Pan, J. K. Gao, E. J. Lv, T. T. Li, H. Xu, L. Sun, A. Nairan, Q. C. Zhang, *Adv.*

Funct. Mater. **2023**, *33*, 2303833.

[33] D. Yoon, J. Lee, B. Seo, B. Kim, H. Baik, S. H. Joo, K. Lee, *Small* **2017**, *13*, 1700052.

[34] Y. Zhu, K. Fan, C. S. Hsu, G. Chen, C. Chen, T. Liu, Z. Lin, S. She, L. Li, H. Zhou, *Adv. Mater.* **2023**, *35*, 2301133.

[35] Y. Zhang, R. Lu, C. Wang, Y. Zhao, L. Qi, *Adv. Funct. Mater.* **2023**, *33*, 2303073.

[36] J. Mahmood, F. Li, S.-M. Jung, M. S. Okyay, I. Ahmad, S.-J. Kim, N. Park, H. Y. Jeong, J.-B. Baek, *Nat. Nanotechnol.* **2017**, *12*, 441.

[37] Y. Kang, O. Cretu, J. Kikkawa, K. Kimoto, H. Nara, A. S. Nugraha, H. Kawamoto, M. Eguchi, T. Liao, Z. Sun, *Nat. Commun.* **2023**, *14*, 4182.

[38] Z. Chen, Y. Xu, D. Ding, G. Song, X. Gan, H. Li, W. Wei, J. Chen, Z. Li, Z. Gong, *Nat. Commun.* **2022**, *13*, 763.

[39] Y. Lu, K. Huang, X. Cao, L. Zhang, T. Wang, D. Peng, B. Zhang, Z. Liu, J. Wu, Y. Zhang, *Adv. Funct. Mater.* **2022**, *32*, 2110645.

[40] X. Bu, Y. Bu, Q. Quan, S. Yang, Y. Meng, D. Chen, Z. Lai, P. Xie, D. Yin, D. Li, *Adv. Funct. Mater.* **2022**, *32*, 2206006.

[41] E. J. Popczun, J. R. McKone, C. G. Read, A. J. Biacchi, A. M. Wiltrout, N. S. Lewis, R. E. Schaak, *J. Am. Chem. Soc.* **2013**, *135*, 9267.

[42] Y. Zhou, T. Lin, X. Luo, Z. Yan, J. Wu, J. Wang, Y. Shen, *J. Catal.* **2020**, *388*, 122.

[43] K. Huang, J. Xia, Y. Lu, B. Zhang, W. Shi, X. Cao, X. Zhang, L. M. Woods, C. Han, C. Chen, *Adv. Sci.* **2023**, *10*, 2300094.

[44] Y. Li, C.-K. Peng, H. Hu, S.-Y. Chen, J.-H. Choi, Y.-G. Lin, J.-M. Lee, *Nat. Commun.* **2022**, *13*, 1143.

[45] Y. Wang, X. Li, M. Zhang, J. Zhang, Z. Chen, X. Zheng, Z. Tian, N. Zhao, X. Han, K. Zaghib, *Adv. Mater.* **2022**, *34*, 2107053.

[46] M. Ju, Z. W. Chen, H. Zhu, R. M. Cai, Z. D. Lin, Y. P. Chen, Y. J. Wang, J. L. Gao, X. Long, S. H. Yang, *J. Am. Chem. Soc.* **2023**, *145*, 11215.

[47] D. Friebel, M. W. Louie, M. Bajdich, K. E. Sanwald, Y. Cai, A. M. Wise, M.-J. Cheng, D. Sokaras, T.-C. Weng, R. Alonso-Mori, *J. Am. Chem. Soc.* **2015**, *137*, 1305.

[48] B. Zhang, X. Zheng, O. Voznyy, R. Comin, M. Bajdich, M. García-Melchor, L. Han, J.

Xu, M. Liu, L. Zheng, *Science* **2016**, *352*, 333.

[49] Y. Zhou, R. Lu, X. Tao, Z. Qiu, G. Chen, J. Yang, Y. Zhao, X. Feng, K. Müllen, *J. Am. Chem. Soc.* **2023**, *145*, 3647.

[50] C. Zhang, Q. Qi, Y. Mei, J. Hu, M. Sun, Y. Zhang, B. Huang, L. Zhang, S. Yang, *Adv. Mater.* **2023**, *35*, 2208904.

[51] M. Q. Yu, L. X. Jiang, H. G. Yang, *Chem. Commun.* **2015**, *51*, 14361.

[52] J. A. Koza, Z. He, A. S. Miller, J. A. Switzer, *Chem. Mater.* **2012**, *24*, 3567.

[53] T. Liu, Y. Liang, Q. Liu, X. Sun, Y. He, A. M. Asiri, *Electrochem. Commun.* **2015**, *60*, 92.

[54] L. Jiao, Y.-X. Zhou, H.-L. Jiang, *Chem. Sci.* **2016**, *7*, 1690.

[55] Y. Liang, Y. Li, H. Wang, J. Zhou, J. Wang, T. Regier, H. Dai, *Nat. Mater.* **2011**, *10*, 780.

[56] S. Mao, Z. Wen, T. Huang, Y. Hou, J. Chen, *Energy Environ. Sci.* **2014**, *7*, 609.

[57] F. Song, X. Hu, *Nat. Commun.* **2014**, *5*, 4477.

[58] L. Trotochaud, J. K. Ranney, K. N. Williams, S. W. Boettcher, *J. Am. Chem. Soc.* **2012**, *134*, 17253.

[59] N. Jiang, B. You, M. Sheng, Y. Sun, *Angew. Chem.* **2015**, *127*, 6349.

[60] B. You, N. Jiang, M. Sheng, S. Gul, J. Yano, Y. Sun, *Chem. Mater.* **2015**, *27*, 7636.

[61] S. Du, Z. Ren, J. Zhang, J. Wu, W. Xi, J. Zhu, H. Fu, *Chem. Commun.* **2015**, *51*, 8066.

[62] J. Lin, P. Wang, H. Wang, C. Li, X. Si, J. Qi, J. Cao, Z. Zhong, W. Fei, J. Feng, *Adv. Sci.* **2019**, *6*, 1900246.

[63] W. Zhu, X. Yue, W. Zhang, S. Yu, Y. Zhang, J. Wang, J. Wang, *Chem. Commun.* **2016**, *52*, 1486.

[64] K. Xu, P. Chen, X. Li, Y. Tong, H. Ding, X. Wu, W. Chu, Z. Peng, C. Wu, Y. Xie, *J. Am. Chem. Soc.* **2015**, *137*, 4119.

[65] Y. Xu, W. Tu, B. Zhang, S. Yin, Y. Huang, M. Kraft, R. Xu, *Adv. Mater.* **2017**, *29*, 1605957.

[66] T. Yoon, K. S. Kim, *Adv. Funct. Mater.* **2016**, *26*, 7386.

[67] Y. F. Cui, S. D. Jiang, Q. Fu, R. Wang, P. Xu, Y. Sui, X. J. Wang, Z. L. Ning, J. F. Sun, X. Sun, *Adv. Funct. Mater.* **2023**, 2306889.

[68] Z. Jin, J. Lyu, Y.-L. Zhao, H. Li, X. Lin, G. Xie, X. Liu, J.-J. Kai, H.-J. Qiu, *ACS Materials Lett.* **2020**, *2*, 1698.

[69] J. Tang, J. Xu, Z. Ye, Y. Ma, X. Li, J. Luo, Y. Huang, *J. Alloys Compd.* **2021**, *885*, 160995.

[70] T. X. Nguyen, Y. H. Su, C. C. Lin, J. M. Ting, *Adv. Funct. Mater.* **2021**, *31*, 2106229.

[71] Z. J. Chen, T. Zhang, X. Y. Gao, Y. J. Huang, X. H. Qin, Y. F. Wang, K. Zhao, X. Peng, C. Zhang, L. Liu, *Adv. Mater.* **2021**, *33*, 2101845.

[72] T. X. Nguyen, Y. H. Su, C. C. Lin, J. Ruan, J. M. Ting, *Adv. Sci.* **2021**, *8*, 2002446.

[73] Q. Wang, J. Li, Y. Li, G. Shao, Z. Jia, B. Shen, *Nano Res.* **2022**, *15*, 8751.

[74] K. Huang, D. Peng, Z. Yao, J. Xia, B. Zhang, H. Liu, Z. Chen, F. Wu, J. Wu, Y. Huang, *Chem. Eng. J.* **2021**, *425*, 131533.

[75] J. Huang, P. Wang, P. Li, H. Yin, D. Wang, *J. Mater. Sci. Technol.* **2021**, *93*, 110.

[76] G. Chen, T. Wang, J. Zhang, P. Liu, H. Sun, X. Zhuang, M. Chen, X. Feng, *Adv. Mater.* **2018**, *30*, 1706279.

[77] H. B. Yang, J. Miao, S.-F. Hung, J. Chen, H. B. Tao, X. Wang, L. Zhang, R. Chen, J. Gao, H. M. Chen, *Sci. Adv.* **2016**, *2*, e1501122.

[78] G. Mu, G. Wang, Q. Huang, Y. Miao, D. Wen, D. Lin, C. Xu, Y. Wan, F. Xie, W. Guo, *Adv. Funct. Mater.* **2023**, *33*, 2211260.

[79] C.-Z. Yuan, S. Wang, K. San Hui, K. Wang, J. Li, H. Gao, C. Zha, X. Zhang, D. A. Dinh, X.-L. Wu, *ACS Catal.* **2023**, *13*, 2462.

[80] Y. J. Mei, Y. B. Feng, C. X. Zhang, Y. Zhang, Q. L. Qi, J. Hu, *ACS Catal.* **2022**, *12*, 10808.

[81] Y. Zhao, X. F. Lu, Z. P. Wu, Z. Pei, D. Luan, X. W. Lou, *Adv. Mater.* **2023**, *35*, 2207888.

[82] Y. Zhang, S. Chen, Y. Zhang, R. Li, B. Zhao, T. Peng, *Adv. Mater.* **2023**, *35*, 2210727.

[83] Y. Wang, R. Yang, Y. Ding, B. Zhang, H. Li, B. Bai, M. Li, Y. Cui, J. Xiao, Z.-S. Wu, *Nat. Commun.* **2023**, *14*, 1412.

[84] J. Zhang, R. Z. Sun, X. F. Zhang, J. X. Wu, Y. H. Dou, X. Y. Zhu, L. H. Yu, L. Y. Guo, M. L. Liu, L. Guo, L. M. Cao, C. T. He, X. M. Chen, *Adv. Funct. Mater.* **2022**, *32*, 2202119.

[85] P. Zhai, C. Wang, Y. Zhao, Y. Zhang, J. Gao, L. Sun, J. Hou, *Nat. Commun.* **2023**, *14*,

1873.

[86] S. Shankar Naik, J. Theerthagiri, F. S. Nogueira, S. J. Lee, A. Min, G.-A. Kim, G. Maia, L. M. Pinto, M. Y. Choi, *ACS Catal.* **2023**, *13*, 1477.

[87] S. Wang, T. Shen, C. Yang, G. Luo, D. Wang, *ACS Catal.* **2023**, *13*, 8670.

[88] Y. Weng, K. Wang, S. Li, Y. Wang, L. Lei, L. Zhuang, Z. Xu, *Adv. Sci.* **2023**, *10*, 2205920.

[89] S. Xu, C. Chen, J. Shen, Z. Xu, Y. Lu, P. Song, W. Dong, R. Fan, M. Shen, *Int. J. Hydrogen Energy* **2023**, *48*, 17882.

[90] H. Ma, Z. Chen, Z. Wang, C. V. Singh, Q. Jiang, *Adv. Sci.* **2022**, *9*, 2105313.

[91] F. Zhang, X. Wang, W. Han, Y. Qian, L. Qiu, Y. He, L. Lei, X. Zhang, *Adv. Funct. Mater.* **2022**, *33*, 2212381.

[92] Y. Chen, G. Meng, Z. Chang, N. Dai, C. Chen, X. Hou, X. Cui, *Nanomaterials* **2022**, *13*, 74.

[93] S. Hao, L. Chen, C. Yu, B. Yang, Z. Li, Y. Hou, L. Lei, X. Zhang, *ACS Energy Lett.* **2019**, *4*, 952.

[94] L. Zeng, K. Sun, X. Wang, Y. Liu, Y. Pan, Z. Liu, D. Cao, Y. Song, S. Liu, C. Liu, *Nano Energy* **2018**, *51*, 26.

[95] B. Zhang, Z. Jiang, X. Shang, S. Li, Z.-J. Jiang, *J. Mater. Chem. A* **2021**, *9*, 25934.

[96] Z. X. Hui, H. Li, Z. W. Chen, Z. Wen, G. Y. Wang, C. V. Singh, C. C. Yang, Q. Jiang, *Small* **2024**, e2407860.

[97] P. Wang, J. Qi, X. Chen, C. Li, W. Li, T. Wang, C. Liang, *ACS Appl. Mater. Interfaces* **2020**, *12*, 4385.

[98] J. Jin, F. Chen, Y. Feng, J. Zhou, W. Lei, F. Gao, *Fuel* **2023**, *332*, 126131.

[99] Q. Lv, L. Yang, W. Wang, S. Lu, T. Wang, L. Cao, B. Dong, *J. Mater. Chem. A* **2019**, *7*, 1196.

[100] Y. Li, S. Guo, T. Jin, Y. Wang, F. Cheng, L. Jiao, *Nano Energy* **2019**, *63*, 103821.

[101] L. Yu, H. Zhou, J. Sun, F. Qin, F. Yu, J. Bao, Y. Yu, S. Chen, Z. Ren, *Energy Environ. Sci.* **2017**, *10*, 1820.

[102] G. Shi, Y. Xie, L. Du, Z. Fan, X. Chen, X. Fu, W. Xie, M. Wang, M. Yuan, *Nano Energy* **2020**, *74*, 104852.

[103] G. Zhang, Y.-S. Feng, W.-T. Lu, D. He, C.-Y. Wang, Y.-K. Li, X.-Y. Wang, F.-F. Cao, *ACS Catal.* **2018**, *8*, 5431.

[104] J. Wang, M. Zhang, G. Yang, W. Song, W. Zhong, X. Wang, M. Wang, T. Sun, Y. Tang, *Adv. Funct. Mater.* **2021**, *31*, 2101532.

[105] Y. Li, H. Zhang, M. Jiang, Q. Zhang, P. He, X. Sun, *Adv. Funct. Mater.* **2017**, *27*, 1702513.

[106] W. Li, Y. Jiang, Y. Li, Q. Gao, W. Shen, Y. Jiang, R. He, M. Li, *Chem. Eng. J.* **2021**, *425*, 130651.

[107] X. Guo, M. Li, L. He, S. Geng, F. Tian, Y. Song, W. Yang, Y. Yu, *Nanoscale* **2021**, *13*, 14179.

[108] A. M. Shah, K. H. Modi, P. M. Pataniya, K. S. Joseph, S. Dabhi, G. R. Bhadu, C. K. Sumesh, *ACS Appl. Mater. Interfaces* **2024**, *16*, 11440.

[109] L. Chen, Y. Song, Y. Liu, L. Xu, J. Qin, Y. Lei, Y. Tang, *J. Energy Chem.* **2020**, *50*, 395.
